# Hsa-miR-143-3p inhibits Wnt-β-catenin and MAPK signaling in human corneal epithelial stem cells

**DOI:** 10.1038/s41598-022-15263-x

**Published:** 2022-07-06

**Authors:** Lavanya Kalaimani, Bharanidharan Devarajan, Venkatesh Prajna Namperumalsamy, Muthukkaruppan Veerappan, Julie T. Daniels, Gowri Priya Chidambaranathan

**Affiliations:** 1 Department of Immunology and Stem Cell Biology, Aravind Medical Research Foundation, Madurai, Tamil Nadu 625020 India; 2 Department of Bioinformatics, Aravind Medical Research Foundation, Madurai, Tamil Nadu India; 3 Cornea Clinic, Aravind Eye Hospital and Postgraduate Institute of Ophthalmology, Madurai, Tamil Nadu India; 4 Department of Biotechnology, Aravind Medical Research Foundation-Affiliated to Alagappa University, Karaikudi, Tamil Nadu India; 5 Institute of Ophthalmology, University College London, London, UK

**Keywords:** Cell biology, Molecular biology, Stem cells

## Abstract

Our previous study demonstrated hsa-miR-143-3p as one of the highly expressed miRNAs in enriched corneal epithelial stem cells (CESCs). Hence this study aims to elucidate the regulatory role of hsa-miR-143-3p in the maintenance of stemness in CESCs. The target genes of hsa-miR-143-3p were predicted and subjected to pathway analysis to select the targets for functional studies. Primary cultured limbal epithelial cells were transfected with hsa-miR-143-3p mimic, inhibitor or scrambled sequence using Lipofectamine 3000. The transfected cells were analysed for (i) colony forming potential, (ii) expression of stem cell (SC) markers/ transcription factors (*ABCG2, NANOG, OCT4, KLF4*, Δ*Np63*), (iii) differentiation marker (Cx43), (iv) predicted five targets of hsa-miR-143-3p (*DVL3, MAPK1, MAPK14, KRAS* and *KAT6A*), (v) MAPK signaling regulators and (vi) Wnt-β-catenin signaling regulators by qPCR, immunofluorescence staining and/or Western blotting. High expression of hsa-miR-143-3p increased the colony forming potential (10.04 ± 1.35%, p < 0.001) with the ability to form holoclone-like colonies in comparison to control (3.33 ± 0.71%). The mimic treated cells had increased expression of SC markers but reduced expression of Cx43 and hsa-miR-143-3p targets involved in Wnt-β-catenin and MAPK signaling pathways. The expression of β-catenin, active β-catenin and ERK2 in hsa-miR-143-3p inhibitor transfected cells were higher than the control cells and the localized nuclear expression indicated the activation of Wnt and MAPK signaling. Thus, the probable association of hsa-miR-143-3p in the maintenance of CESCs through inhibition of Wnt and MAPK signaling pathways was thus indicated.

## Introduction

Cornea and the tear film comprise the anterior transparent window of the eye, which acts as a physical barrier between the optical and external environment. The corneal epithelium is the outer most layer of the cornea and is regenerated from the corneal epithelial stem cells (CESCs) that are present in the basal layer of the limbal epithelium^1^. These adult tissue resident stem cells constitute 3–5% of the total limbal epithelium and are normally quiescent. However, the prime function of CESCs is to govern the processes like wound healing^2^ and homeostasis^3^ to maintain corneal transparency^4^. The molecular mechanism behind the maintenance of stemness in CESCs is still not clear including the epigenetic regulation by microRNAs (miRNAs).

MiRNAs are non-coding single stranded RNAs (18 to 24 nucleotides) and they regulate the protein levels of the target messenger RNA (mRNA) without any modification in gene sequence^5^. MiRNAs are known to actively participate in various cellular processes like proliferation, differentiation, cellular metabolism, homeostasis and apoptosis^6,7^.

In our previous study, CESCs have been enriched to 80% using a two-step protocol^8^ and compared to differentiated central corneal epithelial cells by small RNA sequencing. Six miRNAs- hsa-miR-3168, hsa-miR-21-5p, hsa-miR-143-3p, hsa-miR-150-5p, hsa-miR-10a-5p and hsa-miR-1910-5p were identified to be highly expressed in the enriched CESCs and validated through qPCR. Interestingly, based on the locked nucleic acid *in-situ* hybridization, hsa-miR-143-3p was found be expressed in the basal layer of the limbal epithelium, exclusively in the clusters of small cells indicating its potential association with the CESCs^9^. In continuation, the functional role of hsa-miR-143-3p in CESCs was evaluated in this study.

In mouse embryonic stem cell, hsa-miR-143-3p promoted self-renewal and increased the expression of pluripotency genes such as *OCT4, KLF4* and *ESRRB*^10^. Hsa-miR-143-3p was also known to regulate various cellular processes like differentiation^11^, proliferation^12,13^, migration^14^, apoptosis^15,16^ and cell cycle regulation^17^.

In this study, limbal epithelial cells cultured by both 2D and 3D culture systems were used for the functional studies. The Real Architecture For 3D Tissue (3D RAFTs) are collagen-based hydrogels in which corneal stromal stem cells (CSSCs) are embedded, which serve as a model of corneal stromal stem cell niche^18^. Thus, the limbal epithelial cells cultured on the 3D RAFTs will have close interaction with CSSCs similar to its native environment. The epithelial or endothelial tissue equivalents produced by this technique are suitable for transplantation and used as a model for studying cellular interactions and functional characteristics^19,20^. Elucidating the signaling mechanism behind the regulation of CESCs by hsa-miR-143-3p will pave platform to develop new miRNA-based culture techniques for expanding the CESCs for transplantation and stem cell therapies to treat patients with limbal stem cell deficiency (LSCD).

## Methods

### Sample

The enucleated donor globes not suitable for transplantation and limbal rims received after corneal transplantation (donor age below 73 years) were obtained from Rotary Aravind International Eye Bank (Madurai, India), Moorfields Eye Hospital Lions Eye Bank (London, UK) and Veneto Eye Bank Foundation (Venice, Italy). The non-vascularized tissues with intact limbus were used in the study after thorough examination under stereo binocular microscope. Human donor tissues were handled in accordance with the tenets of the Declaration of Helsinki. The study was approved by Institutional Ethics Committee of Aravind Medical Research Foundation (RES2013038BAS) and the Moorfields Eye Hospital / UCL Institute of Ophthalmology Eye Tissue Repository (10/H0106/57-2011ETR10). Informed consent for the use of donor eyes for research were obtained from the legally authorized representatives.

### Target prediction for hsa-miR-143-3p

The targets of hsa-miR-143-3p were predicted using miRWalk (Version 3.0)^21^ and mirDIP (Version 4.1.1.6)^22^. The targets that were common in both miRWalk and mirDIP were selected to avoid false positives. The selected targets were subjected to analysis by Kyoto Encyclopedia of Genes and Genomes (KEGG) mapping tool, Search pathway in KEGG mapper and grouped into functional categories. The targets associated with regulation of stem cells were selected for further analysis.

### 2D limbal epithelial cell culture

Limbal epithelial cells were cultured from the 2 mm limbal explants dissected from the donor globes or limbal rims. The explants were placed in the supplemented hormonal epithelial medium (SHEM) media^23^ coated 35-mm cell culture dish (Nunc, Thermofisher Scientific, Massachusetts, United States) with epithelial side facing up. The explants were incubated at 37 °C for 20 min to allow for their attachment to the culture dish. The attached explants were then cultured in SHEM at 37 °C and 5% CO_2._ The media was changed on alternate day till they reached 70 to 80% confluency.

### Limbal epithelial cell culture on 3D RAFTs

3D RAFTs containing CSSCs were used for the culture of primary limbal epithelial cells. The limbal epithelial cells were obtained by incubating the limbal rims in dispase II (2 mg/ml) for 45 min at 37 °C. Then the limbal epithelium was scraped off using a sterile scalpel. The total limbal epithelium was subjected to trypsin (0.25%) treatment for 30 min to obtain individual cells^24^.

For CSSC culture, the limbal region together with anterior stroma was dissected and subjected to collagenase-L (0.5 mg/ml) treatment for 16 h at 37 °C. The cells were separated from the solution through centrifugation and the pellet was resuspended in 3 ml of CSSC medium^25^. The resuspended cells were cultured at 37 °C in 5% CO_2_ in fibronectin coated T25 flask for 24 h. The cells were supplemented with fresh CSSC medium after 24 h and the non-adhered cells were removed. By second day, selective trypsinization for CSSCs were carried out with 0.05% Trypsin-0.02% EDTA (Invitrogen) and the CSSCs were seeded in the fresh fibronectin coated T75 flask. The CSSCs were cultured at 37 °C in 5% CO_2_ with media change on every alternate day. At 60–70% confluency, the cells were sub-cultured with the seeding density of 3000 cells/cm^2^ in a fresh flask. For the preparation of 3D-RAFT, the CSSCs at passage 4, validated for the CSSC marker expression by immunostaining were used.

For the preparation of RAFT TEs, the AteloCell Native collagen (Bovine dermis, 3 mg/mL, pH 3.0, collagen acidic solution I-AC) (Koken, Tokyo, Japan) was mixed with 10X Minimum Essential Medium (MEM) from RAFT reagent kit (Lonza, Basel, Switzerland) in the ratio of 8:1. The collagen solution was adjusted to pH between 7.2 and 7.4 using the neutralising solution (5 M Sodium hydroxide)^26^. The solution was centrifuged at 1000 rpm for 3 min. The CSSCs (30,000 cells/ RAFT) were resuspended in the CSSC medium and added to the collagen solution. A volume of 625 µl of freshly prepared collagen solution with CSSCs was transferred to each well of a 24 well plate (Greiner, Stonehouse, UK) and heated to 37 °C for 30 min. Once the collagen gels were formed, RAFT absorbers for 24-well plates (hydrophilic porous absorbers) (Lonza) were applied to the surface of the hydrogels for 30 min. Then the absorbers were gently removed and fresh CSSC media was added to the RAFT TEs and stored at 37 °C. The limbal epithelial cells were seeded in 24 well plate at the density of 2.5 × 10^4^ cells/ RAFT and cultured in SHEM media at 37 °C and 5% CO_2_. The cells were cultured until they reach 70 to 80% confluency with media change on every alternate day.

### miRNA transfection

The transfection of 70–80% confluent human primary limbal epithelial cell cultures grown in both 2D and 3D culture systems were carried out using Lipofectamine 3000 transfection reagent (Thermofisher Scientific) with 25 nM of hsa-miR-143-3p mimic (miScript miRNA Mimic, Qiagen, Hilden, Germany) or inhibitor (miScript miRNA Inhibitor, Qiagen) or transfection control- scrambled sequence (AllStars Negative Control siRNA, Qiagen) following the manufacturer’s instructions. The transfection was carried out for 4 h at 37 oC in 5% CO_2_ incubator and the cells were supplemented with SHEM. After 2 days of culture, the transfected cells were harvested for the following experiments (i) colony forming assay, (ii) immunofluorescence staining, (iii) RNA isolation for qPCR analysis and (iv) protein isolation for Western blotting. The experiments were replicated thrice with three biological samples (n = 3).

### Colony forming assay

The hsa-miR-143-3p mimic or inhibitor or scrambled control transfected cells were seeded on mitomycin C (4 µg/ml, Sigma-Aldrich) inactivated NIH 3T3 mouse fibroblast feeder layer in 60 mm dish independently at a seeding density of 500 cells per plate. After 12 days of culture in SHEM, the feeder layer was removed with 0.02% EDTA solution (Sigma-Aldrich) and the colonies in the dish were stained with 1% Rhodamine B (Roche, Basel, Switzerland) for 30 min after 15 min fixation with 4% paraformaldehyde (Sigma-Aldrich). Colonies were washed with sterile distilled water and images were captured (Nikon D750 camera, Japan). Colony forming efficiency (CFE) was calculated using the formula: number of colonies formed / number of cells seeded × 100. The colonies were termed as holoclone-like with respect to their size and morphology as defined by Barrandon and Green^27^.

### qPCR

The total RNA was isolated using RNeasy mini kit (Qiagen) from the three groups (i) mimic treated, (ii) inhibitor treated and (iii) scrambled treated. High-Capacity cDNA Reverse Transcription Kit (Thermofisher Scientific) was used for reverse transcription of RNA according to manufacturer’s instruction. Subsequent qPCR amplification was performed for 40 cycles using KAPA SYBR FAST qPCR Master Mix (2X) (Sigma-Aldrich). The qPCR cycle conditions were (i) initial activation for 3 min at 95 °C, (ii) 40 cycles of (a) Denaturation for 10 s at 95 °C, (b) annealing for 20 s at 58 °C and (c) extension for 30 s at 72 °C and (iii) final extension for 7 min at 72 °C. Glyceraldehyde 3-phosphate dehydrogenase (*GAPDH*) was used as reference mRNA. The experiment was repeated thrice with the limbal epithelial cells grown by 2D culture system and the data were represented as the mean ± standard deviation (SD) of the expression value. The primers used for qPCR are listed in supplementary table S1.

### Immunofluorescence staining

The transfected cells grown in 2D culture system were trypsinised with TrypLE Express (Gibco-Thermofisher Scientific) after 2 days of culture and cytocentrifuged (400 rpm; 3 min) on the slides (2.5 × 10^4^ cells/slide). The cells were fixed for 20 min with 4% paraformaldehyde at 25 °C. After fixation, the cells were washed with 1X PBS (thrice) and permeabilized with 0.5% Triton X-100 for 10 min at 25 °C. Following permeabilization, cells were washed with 1X PBS and blocked with 5% goat serum (Sigma-Aldrich) for 60 min. The cells were then incubated with primary antibody diluted in 2% goat serum at 4 °C over night (Supplementary Table S2: List of antibodies used for immunostaining). The cells were washed with PBS to remove the unbound antibodies and incubated with appropriate secondary antibody (1:500) conjugated with fluorophore (Alexa Fluor 488 or Alexa Fluor 555) in PBS for 60 min at 25 °C. The immunostained cells were mounted using vectashield medium with DAPI (Vector laboratories Ltd, Peterborough, UK) after thorough washing with PBS and sealed with coverslip. The images were acquired using confocal laser scanning microscope (Zeiss LSM 700, Germany) for analyzing the localization pattern of protein expression. For each primary antibody used, the corresponding isotype control was used as negative control. The experiment was replicated thrice with three biological samples (n = 3). The transfected cells on 3D RAFTs were immunostained directly on the culture plates and they were not subjected to trypsin treatment. For quantification of the protein expression, the images were taken with the fluorescence microscope Axioskop 2 (Zeiss). The marker expression was quantified with histogram function of HCImage analyser software based on the mean intensity of the fluorescence signal in different channels. The relative protein expression based on fluorescence intensity was quantified as described by Lee et al.^28^

### Western blotting

For the isolation of protein from the transfected cells, the cells were lysed with the mixture of RIPA lysis and extraction buffer (Thermo Scientific) and Halt protease inhibitor cocktail (Thermo Scientific) after washing with ice cold PBS (Gibco, Thermo Scientific). The concentration of the isolated protein was estimated using BCA Protein Assay kit (Pierce, Thermo Scientific). Equal amounts of extracted protein from each sample (20 µg) were separated by 10% Bis–tris gel (NuPAGE, Thermo Scientific) under reducing conditions after heating for 10 min at 95 °C along with LDS sample-loading buffer (Thermo Scientific).

The separated proteins were then electro transferred to a PVDF membrane (Invitrolon, Thermo Scientific). The membrane was blocked with 5% skimmed milk in Tris-buffered saline containing 0.1% Tween 20 (TBST) and incubated at 4 °C overnight with primary antibody (Ab) (Supplementary Table S3: List of primary antibodies used for Western blotting). The membranes were washed thrice and incubated with the appropriate horseradish peroxidase–conjugated secondary antibody at 25 °C for 1 h (Cell Signaling Technology, Inc., Massachusetts, United States). The protein bands were detected using enhanced chemiluminescence reagent after thorough washing with TBST (Millipore, Billerica, MA). In every blot, GAPDH was used as normalizing reference and loading control. The experiment was repeated thrice using the limbal epithelial cells grown both in 2D and 3D culture system and the data were represented as the mean ± SD. To analyze multiple proteins from the same blot and to avoid repeated stripping, the membrane was cut based on the molecular weight of the protein to be analyzed, prior to hybridization with antibodies.

### Statistical analysis

Statistical software STATA 14.0 (Texas, USA) was used for the statistical analysis. All the experiments were carried out in triplicates and the data were represented as mean ± SD. Independent t-test (parametric) was performed to compare the two experimental group when the data followed Gaussian distribution and Mann–Whitney U test (non-parametric) was used for the data that followed non-Gaussian distribution based on the Shapiro–Wilk normality test. p < 0.05 was considered to be statistically significant.

### Ethical approval

Approval was obtained from Institutional Ethics Committee, Aravind Medical Research Foundation (RES2013038BAS) and the Moorfields Eye Hospital/UCL Institute of Ophthalmology Eye Tissue Repository 10/H0106/57-2011ETR10. The procedures used in this study adhere to the tenets of the Declaration of Helsinki.

### Informed consent

Informed consent was obtained for all donor eyes including the minors from the legally authorized representative—either the donor’s parents or family through the Eye Banks.

## Results

### Predicted target genes of hsa-miR-143-3p

For hsa-miR-143-3p, miRWalk and mirDIP identified 1229 and 1661 target genes respectively. A total of 276 common target genes were submitted to KEGG mapper and the putative targets genes were grouped into 231 KEGG pathways. The pathways included MAPK pathway, Wnt signaling pathway, signaling pathway associated with pluripotency of stem cells and PI3K-AKT pathway. Dishevelled segment polarity protein 3 (DVL3), Lysine acetyltransferase 6A (KAT6A), Kirsten rat sarcoma 2 viral oncogene homolog (KRAS), Mitogen-activated protein kinase 1 (MAPK1)/ ERK2 and Mitogen-activated protein kinase 14 (MAPK14)/p38 MAPK were the five targets involved in the signaling pathways regulating pluripotency of stem cells and they were selected for further analysis. The KEGG pathway^29^ showing these selected target genes of hsa-miR-143-3p was represented in Fig. S1.

### Downregulation of predicted targets by hsa-miR-143-3p

**Figure 1:**
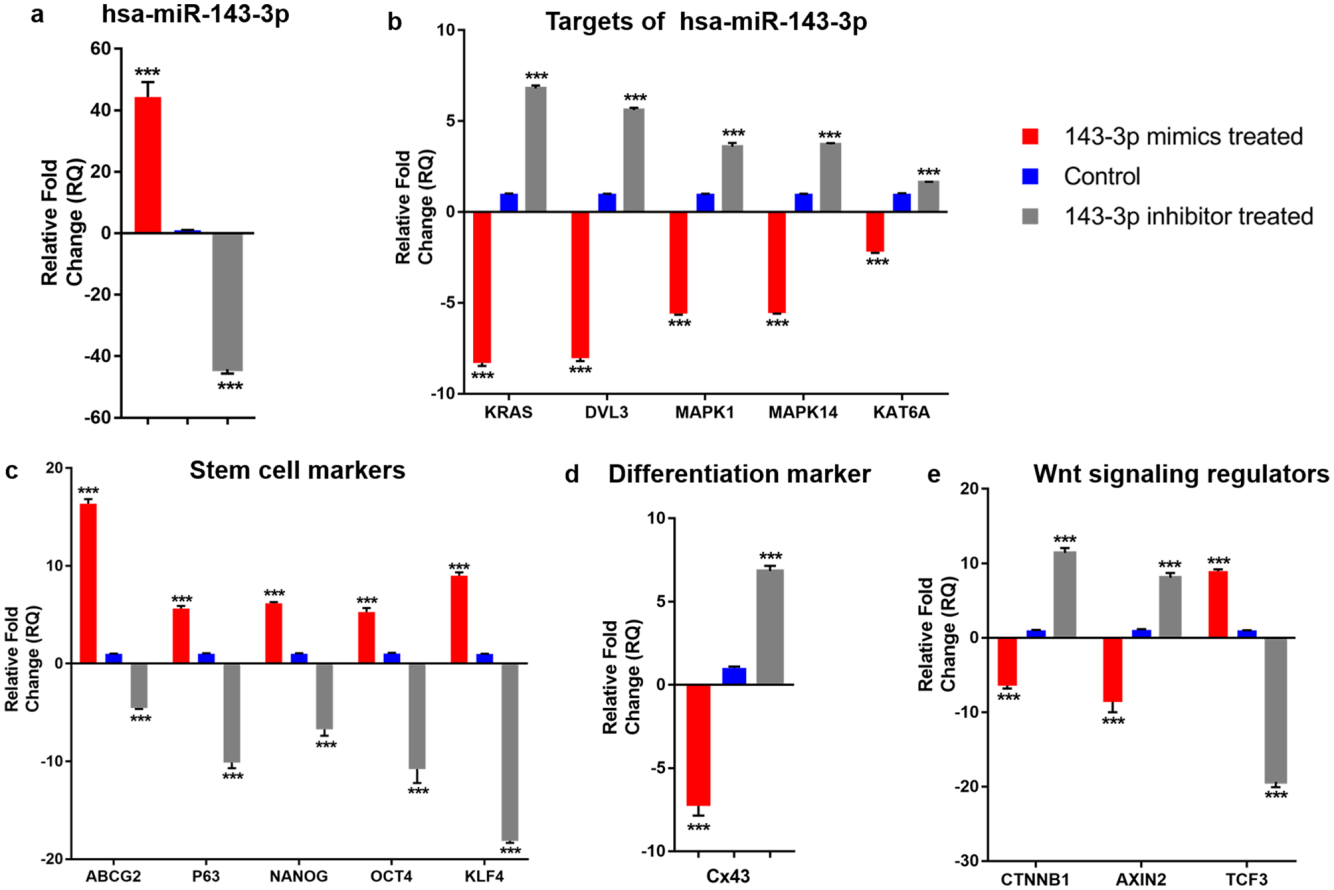
mRNA expression profile of hsa-miR-143-3p transfected cells. Expression profile of (**a**) hsa-miR-143-3p, (**b**) its predicted targets, (**c**) stem cell markers, (**d**) differentiation marker and (**e**) Wnt signaling regulators upon transfection with hsa-miR-143-3p mimic and inhibitor. Relative mRNA/miRNA expression in mimic transfected and inhibitor transfected cells were quantified in comparison to control by qPCR using SYBR Green chemistry. Each sample (n = 3) was run in triplicate. The data were expressed as mean ± SD and relative fold change of expression (RQ) was calculated by 2^−∆∆CT^ method after normalization with GAPDH (reference gene)/ RNU6B (reference miRNA). ***P < 0.0001.

**Figure 2:**
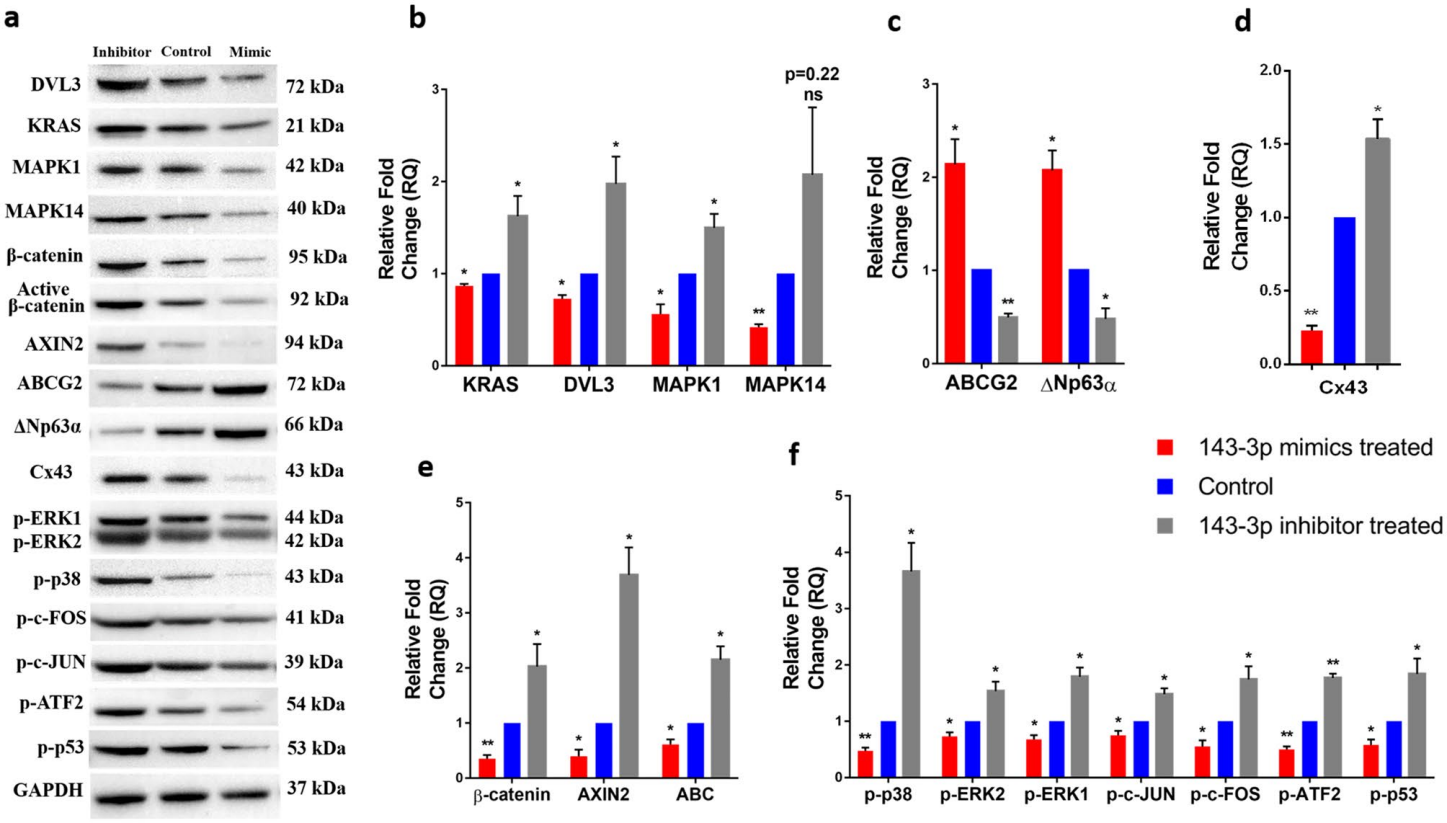
Protein expression profile of hsa-miR-143-3p transfected cells grown in 2D culture system. (**a**) Representative Western blots of protein of interest in three groups-hsa-miR-143-3p mimic, inhibitor and control (n = 3): (i) hsa-miR-143-3p targets: DVL3, KRAS, MAPK1 and MAPK14, (ii) stem cell markers: ABCG2 and ΔNp63α (iii) differentiation marker: Cx43, (iv) Wnt signaling regulators: AXIN2, β-catenin and active β-catenin and (v) MAPK signaling regulators: p-ERK1/2, p-p38, p–c-JUN, p–c-FOS, p-ATF2 and p-p53. GAPDH was used as normalising reference and loading control. Bar graphs indicating the relative expression profile of the proteins quantified by Western blotting (**b**) hsa-miR-143-3p targets (**c**) Stem cell markers (**d**) Differentiation marker (**e**) Wnt signaling regulators (**f**) MAPK signaling regulators. *P < 0.05; **P < 0.001. The relative protein expression values are provided in Table S4 and original Western blots images are provided in Fig. S2.

**Figure 3:**
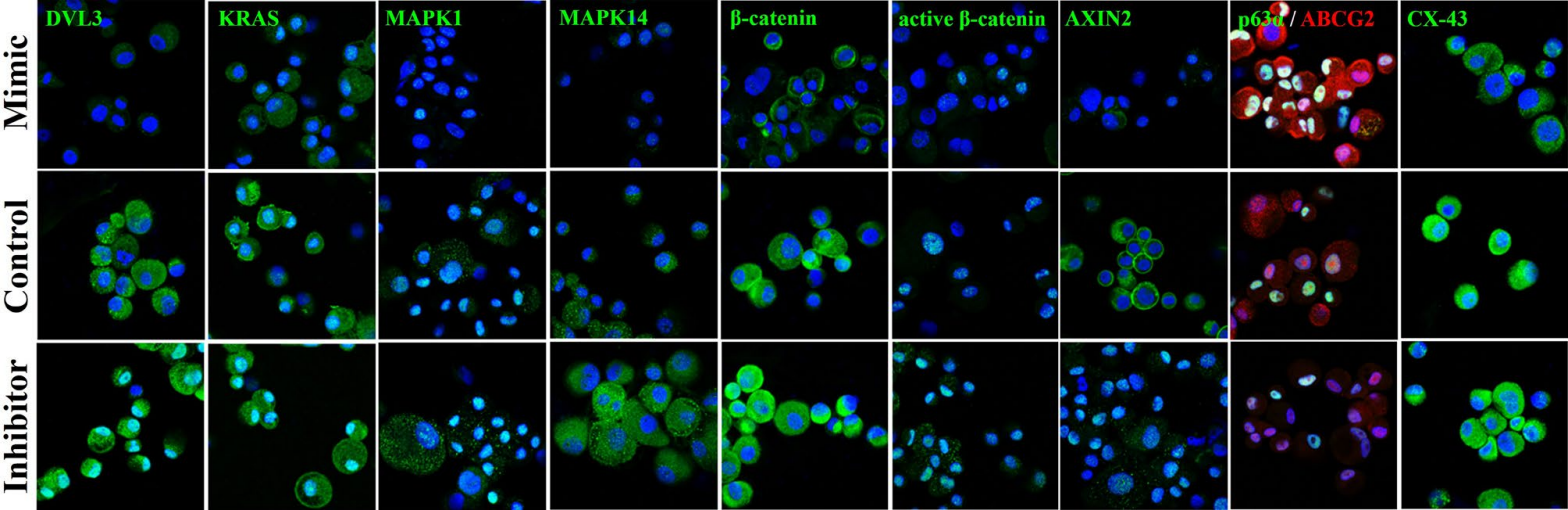
Localization of protein expression in hsa-miR-143-3p transfected cells. Representative confocal images of transfected limbal primary culture cells immunostained for DVL3, KRAS, MAPK1, MAPK14, β-catenin, Active β-catenin, AXIN2, ABCG2, p63⍺ and Cx43. Nuclei were stained with DAPI (Blue) and the protein expression with Alexa Fluor 555 (green) except for ABCG2 with Alexa Fluor 488 (red). Scale bar 50 µm. The detailed split channel images along with their relative fluorescent intensity are provided in Fig. S3.

After transfection, the hsa-miR-143-3p mimic transfected cells showed increased expression of hsa-miR-143-3p (44.35 ± 14.57, p < 0.0001) and inhibitor transfected cells had reduced expression (− 44.19 ± 4.39, p < 0.0001) compared to that of the control cells (Fig. 1A). The expression of the five selected target mRNAs of hsa-miR-143-3p , (i) *DVL3* (− 8.04 ± 0.47, p < 0.0001), (ii) *KAT6A* (− 2.18 ± 0.23, p < 0.0001), (iii) *KRAS* (− 8.31 ± 0.49, p < 0.0001)*,* (iv) *MAPK1* (− 5.59 ± 0.18, p < 0.0001) and (v) *MAPK14* (− 5.56 ± 0.14, p < 0.0001) were downregulated in mimic transfected cells compared to that of the control cells. However, in the inhibitor transfected cells their expression was upregulated significantly (p < 0.0001) (Fig. 1B). Similarly at protein level, the mimic transfected cells had reduced expression of DVL3 (0.73 ± 0.07, p = 0.0024), KRAS (0.87 ± 0.04, p = 0.0037), MAPK1 (0.56 ± 0.18, p = 0.0145) and MAPK14 (0.42 ± 0.06, p = 0.0001) compared to that of the control cells by Western blot (Fig. 2, p < 0.05) and immunostaining (Fig. 3).

### Regulation of stemness and differentiation by hsa-miR-143-3p

At mRNA level, the mimic transfected cells had significantly higher expression of stem cell markers compared to control cells-(i) universal stem cell marker, *ABCG2* (16.36 ± 1.41, p < 0.0001), (ii) limbal stem cell marker, *p63α* (5.65 ± 0.79, p < 0.0001) and (iii) embryonic stem cell markers, *OCT4* (5.28 ± 1.25, p < 0.0001), *NANOG* (6.17 ± 0.41, p < 0.0001) and *KLF4* (9.00 ± 1.00, p < 0.0001) (Fig. 1C). In contrast, the expression of differentiation marker connexin 43 (*Cx43*) was reduced in mimic transfected cells (− 7.27 ± 1.75, p < 0.0001). On the other hand, in the inhibitor transfected cells, the expression of stem cell markers: *ABCG2* (− 4.53 ± 0.34, p < 0.0001), *p63α* (− 10.13 ± 1.69), *OCT4* (− 10.78 ± 4.31, p < 0.0001), *NANOG* (− 6.70 ± 2.00, p < 0.0001), *KLF4* (− 18.13 ± 0.62, p < 0.0001) were reduced and *Cx43* (6.85 ± 0.90, p < 0.0001) expression was increased compared to control cells (Figs. 1D, 3).

Confocal analysis of the mimic transfected cells identified a significant increase in the number of cells (50.29 ± 1.81%, p < 0.0001) which were double positive for ABCG2 and p63α than control (11.96 ± 3.07%, p = 0.0025). In inhibitor transfected cells, there were no cells which express both ABCG2 and p63α (Fig. 3).

The Western blot analysis of stem cell markers and differentiation marker in the transfected cells reavealed that the level of expression of ABCG2 (2.14 ± 0.47, p = 0.0134) and ΔNp63α (2.07 ± 0.37, p = 0.0074) were upregulated and Cx43 expression (0.23 ± 0.05, p < 0.0001) was downregulated in mimic transfected cells (Fig. 2).

**Figure 4:**
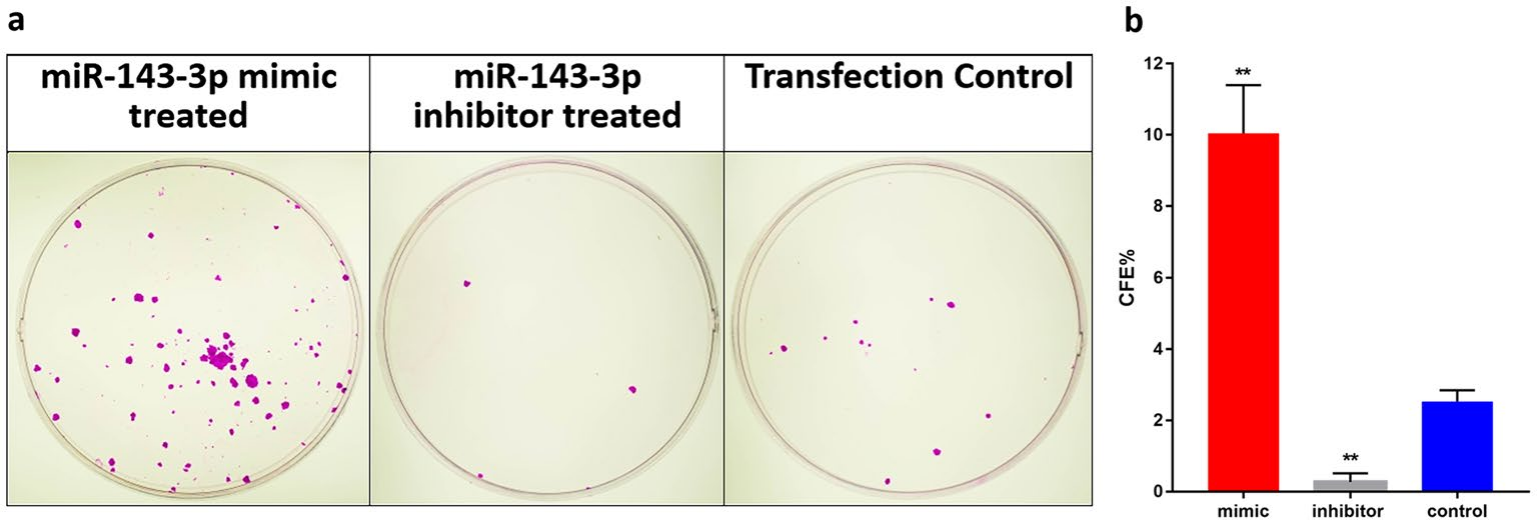
Colony forming potential of hsa-miR-143-3p transfected cells. (**a**) Representative Rhodamine B stained limbal epithelial cell colonies after 12 days of culture in the three groups: hsa-miR-143-3p mimic, inhibitor and control transfected cells. (**b**) The bar graph representing the colony forming efficiency of the cells in the three groups. **P < 0.001.

To confirm the presence of stem cells in transfected cultures, colony forming assay was carried out. The mimic treated cells showed increased percentage of colony forming efficiency (10.04 ± 0.45, p = 0.0003) compared to that of control (3.33 ± 0.23) and inhibitor treated cells (0.26 ± 0.08, p = 0.0003). In addition, there was a significant increase in the percentage of holoclone-like colonies (7.66 ± 2.45, p < 0.05) compared to that of the control (0.69 ± 2.08). The inhibitor treated cells produced no such larger colony (Fig. 4). Thus, the higher expression of hsa-miR-143-3p increased the colony forming efficiency and supported holoclone-like colony formation.

### Effect of hsa-miR-143-3p on Wnt- β-catenin and MAPK signaling

The expression of Wnt signaling regulators *CTNNB1* (− 6.44 ± 1.14, p < 0.0001) and *AXIN2* − 8.64 ± 4.06, p < 0.0001) was downregulated and Wnt signaling transcriptional repressor *TCF3* (8.96 ± 0.71, p < 0.0001) was upregulated in mimic transfected group compared to that of control (Fig. 1E) whereas it was reverse in case of inhibitor transfected cells.

At protein level, immunostaining (Fig. 3) and Western blots revealed that the expression of β-catenin (0.35 ± 0.12, p = 0.0006), active β-catenin (0.61 ± 0.16, p = 0.0140) and AXIN2 (0.39 ± 0.21, p = 0.0075) were reduced in mimic transfected cells. In inhibitor transfected cells the expression of β-catenin (2.00 ± 0.62, p = 0.0493), active β-catenin (2.15 ± 0.43, p = 0.0095) and AXIN2 (3.69 ± 0.86, p = 0.0057) were increased compared to that of the control (Fig. 2, p < 0.05).

The MAPK signaling regulators p-ERK1/2 (0.67 ± 0.13, p = 0.0128; 0.73 ± 0.12, p = 0.0183), p-p38 (0.47 ± 0.10, p = 0.0007), p–c-JUN (0.75 ± 0.14, p = 0.0342), p–c-FOS (0.55 ± 0.18, p = 0.0140), p-ATF2 (0.50 ± 0.09, p = 0.0008) and p-p53 (0.58 ± 0.17, p = 0.0115) were down regulated in mimic transfected cells. However, the expression of MAPK signaling regulators was upregulated in inhibitor transfected cells (Fig. 2, p < 0.05).

### Effect of hsa-miR-143-3p on primary limbal epithelial cells cultured on 3D RAFT system

**Figure 5:**
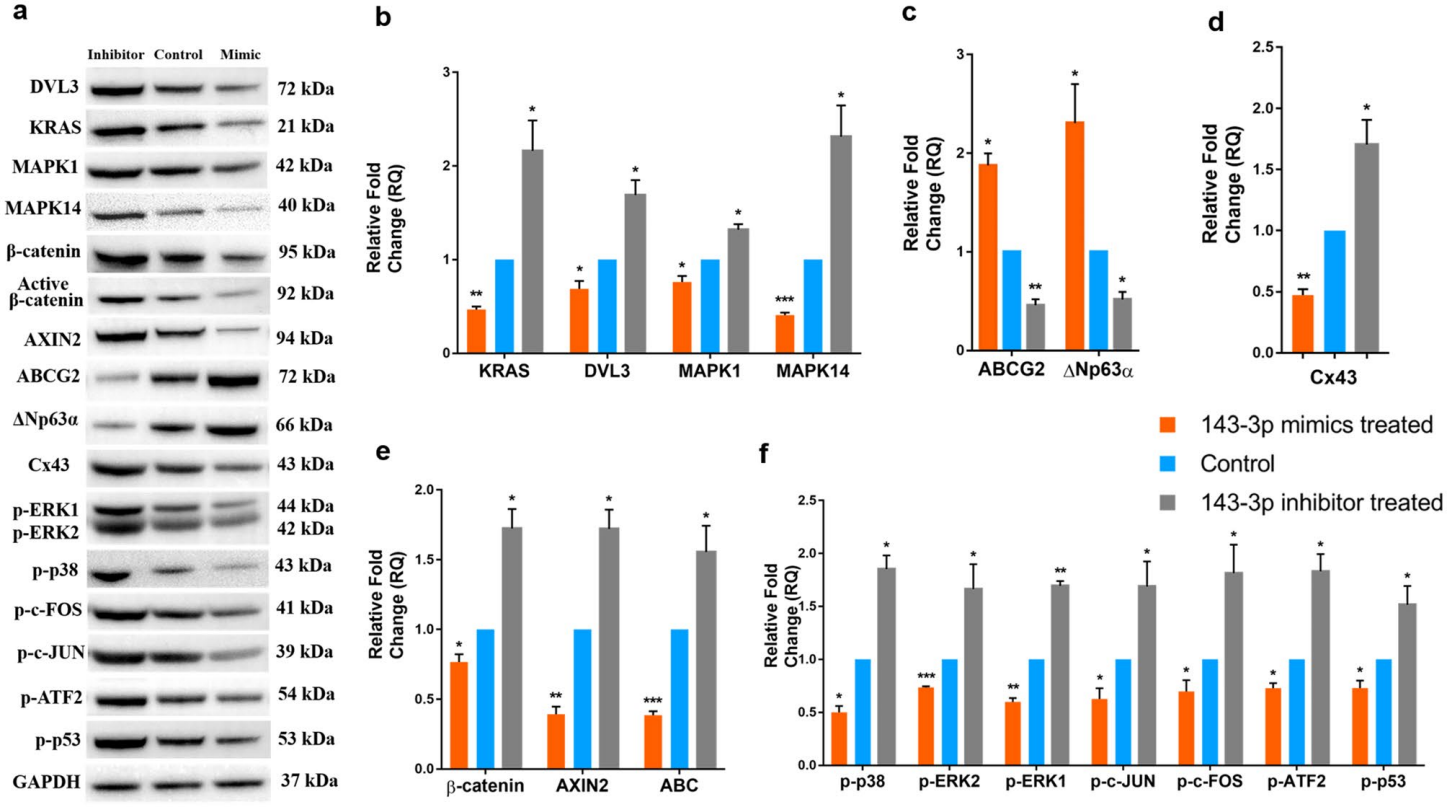
Protein expression profile of hsa-miR-143-3p transfected cells grown in 3D RAFT culture system. (**a**) Representative Western blots of protein of interest in three groups-hsa-miR-143-3p mimic, inhibitor and control transfected 3D RAFT cultured cells (n = 3): (i) hsa-miR-143-3p targets: DVL3, KRAS, MAPK1 and MAPK14, (ii) stem cell markers: ABCG2 and ΔNp63α (iii) differentiation marker, Cx43, (iv) Wnt signaling regulators: AXIN2, β-catenin and active β-catenin and (v) MAPK signaling regulators: p-ERK1/2, p-p38, p–c-JUN, p–c-FOS, p-ATF2 and p-p53. GAPDH was used as normalising reference and loading control. Bar graphs indicating the relative expression profile of the proteins quantified by Western blotting (**b**) hsa-miR-143-3p targets (**c**) Stem cell markers (**d**) Differentiation marker (**e**) Wnt signaling regulators (**f**) MAPK signaling regulators. *P < 0.05; **P < 0.001; ***P < 0.0001. The relative protein expression values are provided in Table S5 and original Western blots images are provided in Fig. S4.

**Figure 6:**
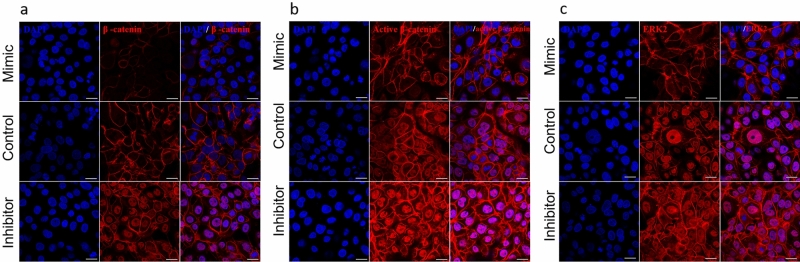
Localization of β-catenin, active β-catenin and ERK2 expression in hsa-miR-143-3p transfected cells in 3D RAFT. Representative confocal images of transfected primary limbal epithelial cells grown on 3D RAFT TEs immunostained for (**a**) β-catenin, (**b**) active- β-catenin and (**c**) ERK2. The nuclei were stained with DAPI (blue) and protein expression with Alexa Fluor 555 (red). Compared to the control and mimic transfected cells, the expression of β-catenin, active-β-catenin and ERK2 were higher in inhibitor transfected cells along with strong nuclear translocation. The relative protein expression values based on fluorescent intensity are provided in Fig. S5. Scale bar 50 µm. Nuclear localization of active-β-catenin and ERK2 indicates the activation of Wnt-β-catenin and MAPK signaling respectively.

The primary limbal epithelial cells grown on the 3D RAFT TEs, upon transfection with mimics had reduced expression of (i) hsa-miR-143-3p targets: DVL3 (0.69 ± 0.15, p = 0.0213), KRAS (0.47 ± 0.05, p = 0.0001), MAPK1 (0.76 ± 0.11, p = 0.0207) and MAPK14 (0.41 ± 0.05, p < 0.0001), (ii) differentiation marker: Cx43 (0.47 ± 0.08, p = 0.0004), (iii) Wnt signaling regulators: AXIN2 (0.39 ± 0.09, p = 0.0004), β-catenin (0.77 ± 0.09, p = 0.0135) and active β-catenin (0.39 ± 0.05, p < 0.0001) and (iv) MAPK signaling regulators:p-ERK1/2 (0.60 ± 0.06, p = 0.0004; 0.74 ± 0.02, p < 0.0001), p-p38 (0.50 ± 0.10, p = 0.0011), p–c-JUN (0.63 ± 0.17, p = 0.0211), p–c-FOS (0.70 ± 0.18, p = 0.0466), p-ATF2 (0.73 ± 0.08, p = 0.0044) and p-p53 (0.73 ± 0.12, p = 0.0193). However, the expression of stem cell markers ABCG2 (1.88 ± 0.21, p = 0.0019) and ΔNp63α (2.31 ± 0.68, p = 0.0299) were increased in mimic transfected cells compared to that of the control (Fig. 5, p < 0.05). In the mimic transfected cells, β-catenin, active β-catenin and ERK2 positivity was confined to the cellular membrane. But in the inhibitor transfected cells, the positivity was observed both in membrane and nucleus indicating nuclear translocation (Fig. 6).

## Discussion

Regulatory role for miRNAs has been identified in various stem cells recently. MiRNAs modulate signaling pathways through selective targeting of the molecules involved^30,31^. For the maintenance of hematopoietic stem cells, miR-143 downregulated Smad-dependent TGFβ/DAB2 signaling^32^. In bone marrow mesenchymal stem cells and apical papillary stem cells, miR-143-3p negatively regulated the differentiation process through targeting bone morphogenetic protein 2^33^ and nuclear factor I-C^34^ respectively. In contrast, it supported differentiation in human dental pulp stem cells via targeting Osteoprotegerin-RANK signaling pathway^35^. Though the expression of hsa-miR-143-3p was identified in various ocular tissues^11,36–38^, its functional association has not been explored.

Our previous study on miRNA profiling of enriched CESCs^9^ suggested the possible role of hsa-miR-143-3p in the maintenance of stemness in CESCs. In continuation, the ectopic expression of hsa-miR-143-3p in this study, increased the colony forming potential of cultured limbal epithelial cells along with increased number of holoclone-like colonies based on the size and morphology. In addition, the expression of stem cell markers was increased and expression of differentiation marker was decreased in the mimic transfected cells. Thus, these results collectively indicated the association of hsa-miR-143-3p in the maintenance of stemness in CESCs.

Association of Wnt and MAPK signaling in the stem cell maintenance as well as differentiation has been reported. In limbal explant cultures, upon inhibition of Wnt signaling^28^ and MAPK signaling^39,40^, the expression of stem cell markers (ABCG2 and p63α) and colony forming efficiency were increased. However, in the suspension culture of isolated limbal epithelial cells Wnt signaling^41^ and MAPK signaling^42^ activation led to reduced colony forming efficiency and number of cells expressing high levels of p63α in cultured limbal colonies.

The mimic transfected cells exhibited reduced expression of four predicted targets of hsa-miR-143-3p: KRAS, MAPK1 (ERK2), MAPK14 (p38α) and DVL3. Though the predicted targets were downregulated, the study has not validated if these targets are the direct targets of hsa-miR-143-3p. Among the targets KRAS was reported to be a known direct target of miR-143-3p^43–45^. The regulatory role of hsa-miR-143-3p’s predicted targets in Wnt and MAPK signaling has been tabulated (Table 1). The expression of Wnt signaling regulators: AXIN2, β-catenin, active β-catenin and MAPK signaling regulators:p-ERK1/2, p-p38, p–c-JUN, p–c-FOS, p-ATF2, p-p53 were downregulated in mimic transfected cells indicating the inhibition of Wnt-β-catenin and MAPK signaling.

**Table 1:** Role of the predicted targets of hsa-miR-143-3p in Wnt and MAPK signaling.

Target mRNA	Signaling	Role
KRAS	Wnt	(i) Activates signaling through inhibition of GSK3β^46^, facilitating accumulation of β-catenin^47^ (ii) Phosphorylates β-catenin at adherens junction leading to its release and stabilization in the cytoplasm, thereby activating Wnt β-catenin signaling^48^
	MAPK	GTP bound KRAS is essential for the activation of ERK-MAPK signaling pathway^49^, downregulation of KRAS inhibits activation of the pathway^50^
MAPK1(ERK2)	Wnt	Activates Wnt signaling through phosphorylation of WNT co-receptor molecule, LDL-related protein 6 (LRP6)^51^
	MAPK	Translocation of ERK2 to the nucleus activates the signaling process^52^, downregulation of ERK2 inhibits MAPK-ERK signaling^53^
MAPK14(p38α)	Wnt	Activates Wnt-β-catenin signaling through inhibition of GSK3β^54^
	MAPK	Accumulation of p-p38 in the cytosol and nucleus is essential for the activation p38-MAPK signaling ^55^
DVL3	Wnt	(i) Upon binding with LRP5/6^56^, DVL3 co-polymerize with AXIN to form the signalosome, leading to the degradation of destruction complex, activating Wnt-β-catenin signaling^57^ (ii) Activates Wnt-β-catenin signaling through phosphorylation of p38^58^
	MAPK	Initiates JNK MAPK signaling through activation of Rho and Rac small GTPases^59^

**Figure 7:**
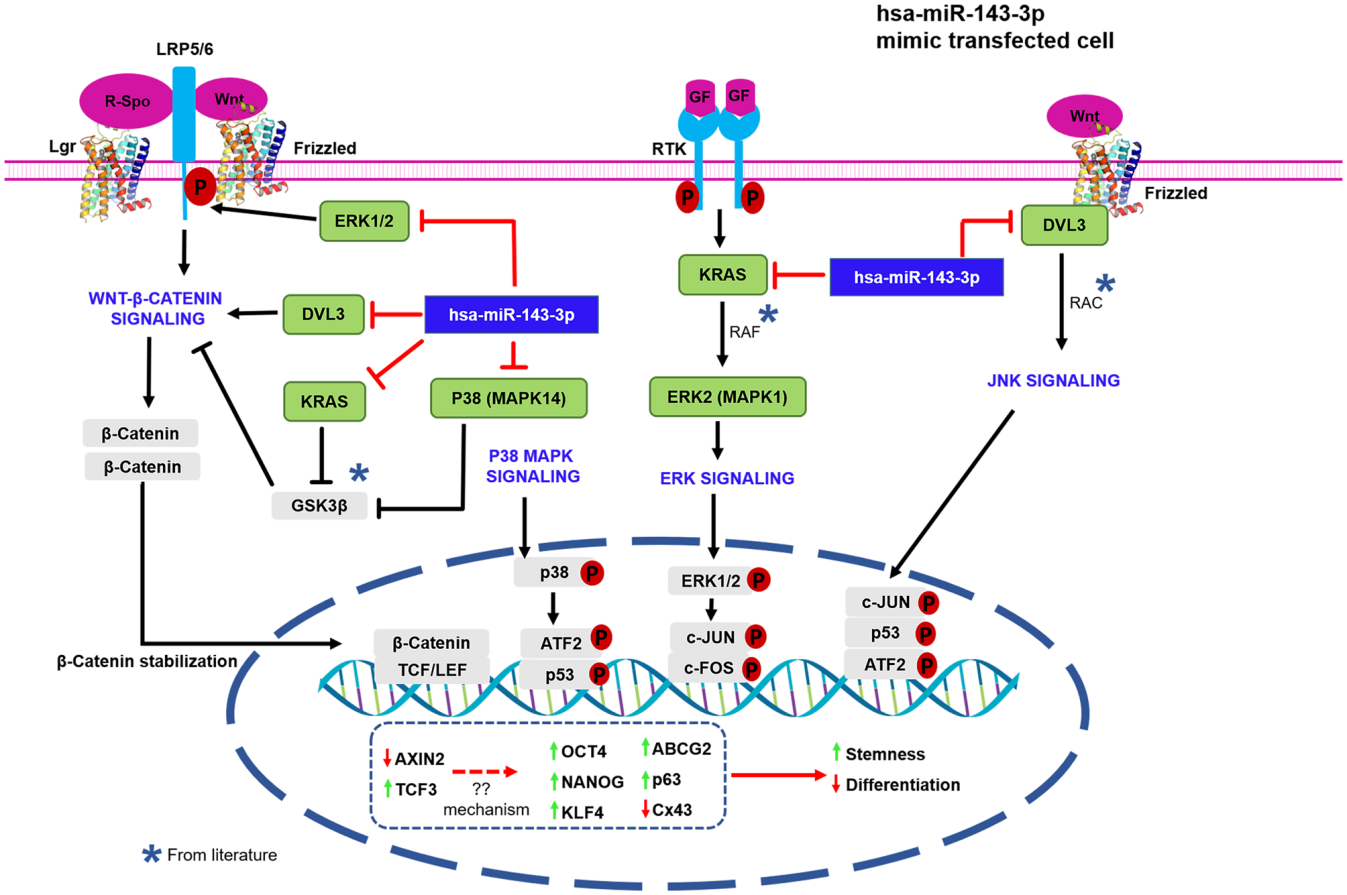
Postulated mechanism of regulation of Wnt and MAPK signaling by hsa-miR-143-3p. Hsa-miR-143-3p inhibits Wnt signaling by downregulating the expression of its targets (i) MAPK14 (p38) and KRAS-inhibits GSK3β, a repressor of Wnt signaling, (ii) DVL3- activates Wnt signaling by dissociating the destruction complex and (iii) MAPK1 (ERK2)- activates the Wnt co-receptor LRP6 through phosphorylation and initiates Wnt signaling. Hsa-miR-143-3p inhibits MAPK signaling through downregulation of its targets (i) DVL3- initiates JNK MAPK signaling through activation of RAC protein (ii) KRAS- activates ERK MAPK signaling through activation of RAF protein (iii) MAPK14- activator of p38-MAPK signaling and (iv) MAPK1-activator of ERK MAPK signaling.

Based on the above observations, we hypothesized a probable mechanism of action of hsa-miR-143-3p on Wnt and MAPK signaling which has been summarized in Fig. 7.

Hsa-miR-143-3p inhibits Wnt signaling by downregulating the expression of its targets (i) MAPK14 (p38)–inhibits GSK3β, a repressor of Wnt signaling (ii) DVL3-activates Wnt signaling by dissociating the destruction complex and by initiating p38 mediated GSK3β inhibition (iii) MAPK1 (ERK2)-activates the Wnt co-receptor LRP6 through phosphorylation and thereby facililating Wnt signaling and (iv) KRAS inhibits GSK3β as well as stabilizes the β-catenin through direct phosphorylation.

Hsa-miR-143-3p inhibits MAPK signaling by downregulating the expression of its targets (i) MAPK14-activation of MAPK14 is the essential step and which marks the activation of p38 MAPK signaling, (ii) DVL3-initiates JNK MAPK signaling through activation of RAC protein, (iii) MAPK1–activation and translocation of MAPK1 to the nucleus is the crucial step in the activation of ERK MAPK signaling and (iv) KRAS-activates ERK MAPK signaling through activation of RAF protein.

## Conclusion

The probable association of hsa-miR-143-3p in the maintenance of CESCs through downregulation of key proteins involved in Wnt and MAPK signaling has been demonstrated in this study using primary limbal epithelial cells grown in 2D and 3D culture systems. Further functional studies are essential to confirm whether the predicted genes are the direct targets of hsa-miR-143-3p and their role in the maintenance of stemness.

## Supplementary Information

Supplementary Information.

## Data Availability

The dataset generated and analysed during the current study are available in the figshare repository, https://doi.org/10.6084/m9.figshare.19242561.v1
