# Supplementary material for: Hsa-miR-143-3p inhibits Wnt-β-catenin and MAPK signaling in human corneal epithelial stem cells
Source: Sci Rep. 2022 Jul 6;12:11432. doi: 10.1038/s41598-022-15263-x (PMC9259643; doi:10.1038/s41598-022-15263-x)
Supplement: Supplementary file 1 — Supplementary Information. [file 41598_2022_15263_MOESM1_ESM.pdf]

Supplementary information

Title: Hsa-miR-143-3p inhibits Wnt-β-catenin and MAPK signaling in human corneal epithelial stem cells

Authors: Lavanya Kalaimani[1,4,5], Bharanidharan Devarajan[2], Venkatesh Prajna Namperumalsamy[3], Muthukkaruppan Veerappan[1], Julie T. Daniels[5], Gowri Priya Chidambaranathan[1,4*]

Table S1: List of primers used in qPCR

Gene name	Accession number	Primer sequence (5' to 3')	Amplicon size
CTNNB1	NM_001330729	Fwd: TCTGAGGACAAGCCACAAGATTACA<br>Rev: TGGGCACCAATATCAAGTCCAA	122 bp
KRAS	NM_001369786	Fwd: GCCTGCTGAAAATGACTGAATATA<br>Rev: TTAGCTGTATCGTCAAGGCACTC	81 bp
MAPK14	NM_139013	Fwd: CCCGAGCGTTACCAGAACC<br>Rev: TCGCATGAATGATGGACTGAAAT	136 bp
MAPK1	NM_138957	Fwd: TTCCAACCTGCTGCTCAACA<br>Rev: TCTGTCAGGAACCCTGTGTGAT	102 bp
DVL3	NM_004423	Fwd: ACAATGCCAAGCTACCATGCTTC<br>Rev: AGCTCCGATGGGTTATCAGCAC	109 bp
KAT6A	NM_001305878	Fwd: CTTACACGGATGCCAAAAGG<br>Rev: GTTTTATCTGTGCCGCCTTC	97 bp
AXIN2	NM_001363813.1	Fwd: GAGTGGACTTGTGCCGACTTCA<br>Rev: GGTGGCTGGTGCAAAGACATAG	189 bp
TCF3	NM_013342	Fwd: GCGTTGCCTCCCCTATTTG<br>Rev: CTGCGATTTAATTCCCGCTGG	161 bp
ΔNp63	NM_001329149	Fwd: AAGAAAGGACAGCAGCATTGAT<br>Rev: GGGACTGGTGGACGAGGAG	155 bp
ABCG2	NM_004827	Fwd: GCACTCTGACGGTGAGAGAAAA<br>Rev: CCAGACACACCACGGATAAACT	169 bp
NANOG	NM_001297698	Fwd: TTCCTTCCTCCATGGATCTG<br>Rev: AAGTGGGTTGTTTGCCTTTG	75 bp
KLF4	NM_001314052	Fwd: CCCACATGAAGCGACTTCCC<br>Rev:CAGGTCCAGGAGATCGTTGAA	170 bp
OCT4	NM_002701	Fwd: GTGTTCAGCCAAAAGACCATCT<br>Rev: GGCCTGCATGAGGGTTTCT	156 bp
Cx43	NM_000165	Fwd: CCTTCTTGCTGATCCAGTGGTAC<br>Rev: ACCAAGGACACCACCAGCAT	154 bp
GAPDH	NM_001357943	Fwd: GTCTCCTCTGACTTCAACAGCG<br>Rev: ACCACCCTGTTGCTGTAGCCAA	131 bp

Fwd- forward; Rev- reverse

Table S2: List of primary antibodies used for immunostaining

Primary antibody	Dilution	Details
K-Ras	1:50	415700 (Invitrogen)
p38 MAPKα	1:100	702273 (Invitrogen)
MAPK1	1:100	MA1-099 (Invitrogen)
DVL-3	1:20	PA5-35096 (Invitrogen)
β-Catenin	1:200	ab16051 (Abcam)
Active β-Catenin	1:200	05-665 (Merck Millipore)
AXIN2	1:20	MAB6078 (R&D Systems)
Cx43	1:100	C6219 (Sigma)
ABCG2	1:20	MAB4146 (Merck Millipore)
p63α	1:100	4892 (Cell Signaling)

Table S3: List of primary antibodies used for Western blotting

Primary antibody	Dilution	Details
K-Ras	1:250	415700 (Invitrogen)
p38 MAPKα (MAPK14)	1:250	702273 (Invitrogen)
Phospho-p38 MAPK	1:250	4511T (Cell Signaling)
MAPK1	1:1000	MA1-099 (Invitrogen)
Phospho-p44/42 MAPK (ERK1/2)	1:250	4370T (Cell Signaling)
DVL-3	1:50	PA5-35096 (Invitrogen)
β-Catenin	1:2000	Ab16051 (Abcam)
Active β-Catenin	1:250	05-665 (Merck Millipore)
AXIN2	1:500	ab32197 (Abcam)
Cx43	1:2000	C6219 (Sigma)
ABCG2	1:50	ab108312 (Abcam)
ΔNp63	1:100	619001 (Biolegend)
GAPDH	1:3000	ab9485 (Abcam)
Phospho-p53	1:250	9284T (Cell Signaling)

Phospho-c-Jun	1:250	ab32385 (Abcam)
Phospho-c-Fos	1:250	ab27793 (Abcam)
Phospho-ATF2	1:250	ab131106 (Abcam)

Table S4 Relative protein expression values in hsa-miR-143-3p transfected cells cultured in 2D by Western blotting

Protein	Expression value		P-value*	Expression value	P-value^
	Control	Mimic		Inhibitor	
KRAS	1.00	0.87 ± 0.04	0.0037	1.62 ± 0.38	0.0470
DVL3	1.00	0.73 ± 0.07	0.0024	1.97 ± 0.51	0.0304
MAPK1	1.00	0.56 ± 0.18	0.0145	1.49 ± 0.27	0.0325
MAPK14	1.00	0.42 ± 0.06	0.0001	2.07 ± 1.27	0.2162
ABCG2	1.00	2.14 ± 0.47	0.0134	0.49 ± 0.07	0.0003
ΔNp63α	1.00	2.07 ± 0.37	0.0074	0.48 ± 0.20	0.0107
Cx43	1.00	0.23 ± 0.05	<0.0001	1.54 ± 0.23	0.0152
AXIN2	1.00	0.39 ± 0.21	0.0075	3.69 ± 0.86	0.0057
β-catenin	1.00	0.35 ± 0.12	0.0006	2.00 ± 0.62	0.0493
Active β-catenin	1.00	0.61 ± 0.16	0.0140	2.15 ± 0.43	0.0095
p-ERK1	1.00	0.67 ± 0.13	0.0128	1.79 ± 0.28	0.0087
p-ERK2	1.00	0.73 ± 0.12	0.0183	1.53 ± 030	0.0360
p-MAPK14	1.00	0.47 ± 0.10	0.0007	3.66±0.88	0.0063
p-c-JUN	1.00	0.75 ± 0.14	0.0342	1.48 ± 0.18	0.0111
p-c-FOS	1.00	0.55 ± 0.18	0.0140	1.74 ± 0.40	0.0333
p-ATF2	1.00	0.50 ± 0.09	0.0008	1.77 ± 0.13	0.0006
p-p53	1.00	0.58 ± 0.17	0.0115	1.84 ± 0.48	0.0378

***Comparison between Control and Mimic group
^Comparison between Control and Inhibitor group

Table S5 Relative protein expression values in hsa-miR-143-3p transfected cells cultured on 3D-RAFT TEs by Western blotting

Protein	Expression value		P-value*	Expression value	P-value^
	Control	Mimic		Inhibitor	
KRAS	1.00	0.47±0.05	0.0001	2.16±0.56	0.0234
DVL3	1.00	0.69±0.15	0.0213	1.69±0.28	0.0124
MAPK1	1.00	0.76±0.11	0.0207	1.32±0.10	0.0054
MAPK14	1.00	0.41±0.05	<0.0001	2.31±0.56	0.0172
ABCG2	1.00	1.88±0.21	0.0019	0.46±0.10	0.0008
ΔNp63α	1.00	2.31±0.68	0.0299	0.52±0.13	0.0032
Cx43	1.00	0.47±0.08	0.0004	1.70±0.35	0.0256
AXIN2	1.00	0.39±0.09	0.0004	1.72±0.24	0.0065
β-catenin	1.00	0.77±0.09	0.0135	1.72±0.24	0.0065
Active β-catenin	1.00	0.39±0.05	<0.0001	1.56±0.3	0.0414
p-ERK1	1.00	0.60±0.06	0.0004	1.69±0.08	0.0001
p-ERK2	1.00	0.74±0.02	<0.0001	1.66±0.40	0.0462
p-MAPK14	1.00	0.50±0.10	0.0011	1.85±0.22	0.0028
p-c-JUN	1.00	0.63±0.17	0.0211	1.69±0.41	0.0433
P-c-FOS	1.00	0.70±0.18	0.0466	1.81±0.46	0.0385
p-ATF2	1.00	0.73±0.08	0.0044	1.83±0.28	0.0069
p-p53	1.00	0.73±0.12	0.0193	1.51±0.31	0.0436

*****Comparison between Control and Mimic group
^Comparison between Control and Inhibitor group

Figures:

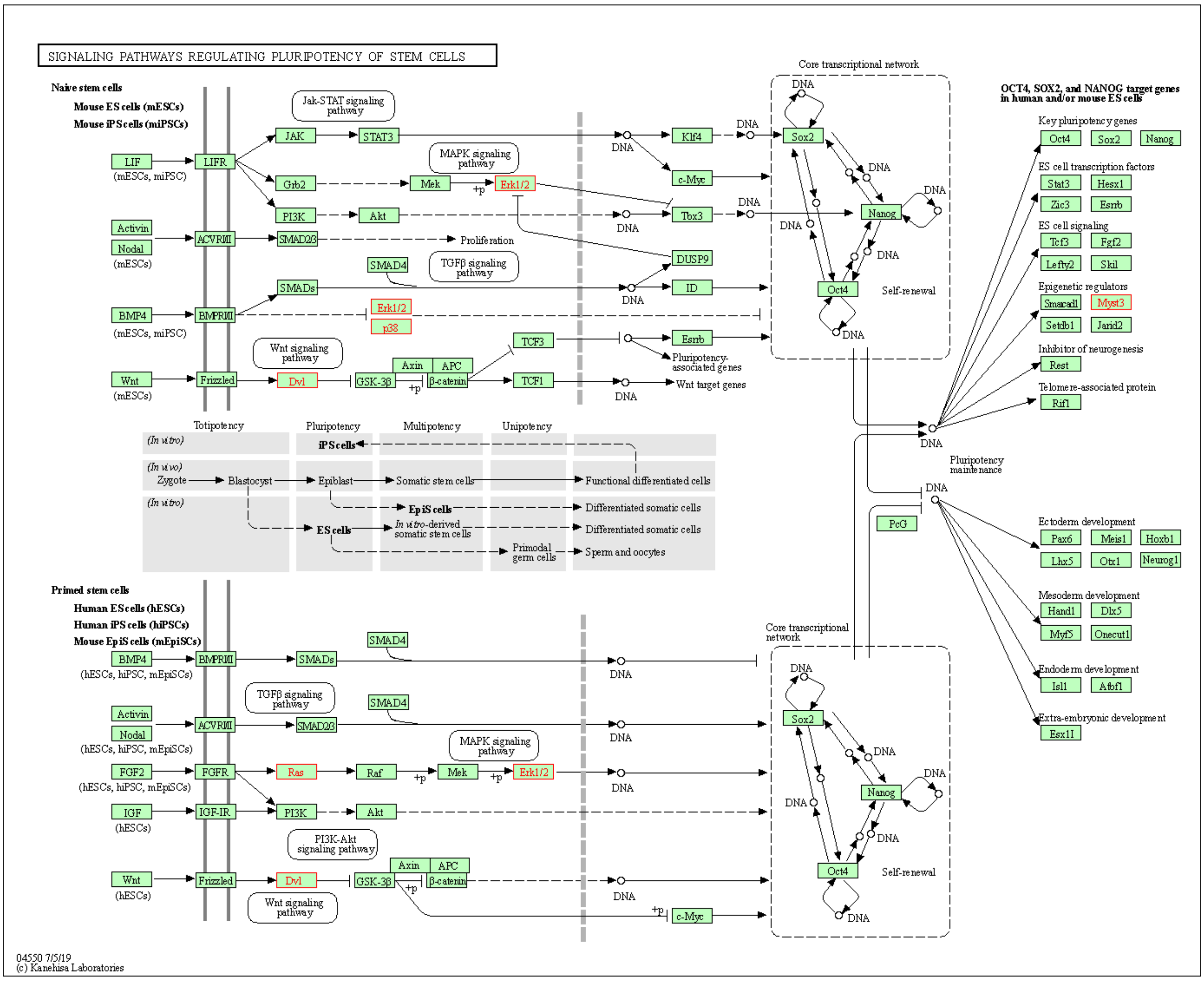

Fig. S1 Pathway analysis of hsa-miR-143-3p predicted targets

The figure represents the KEGG pathway analysis of hsa-miR-143-3p predicted targets associated with the pathways regulating pluripotency of stem cells. The targets of hsa-miR-143-3p are represented in red box.

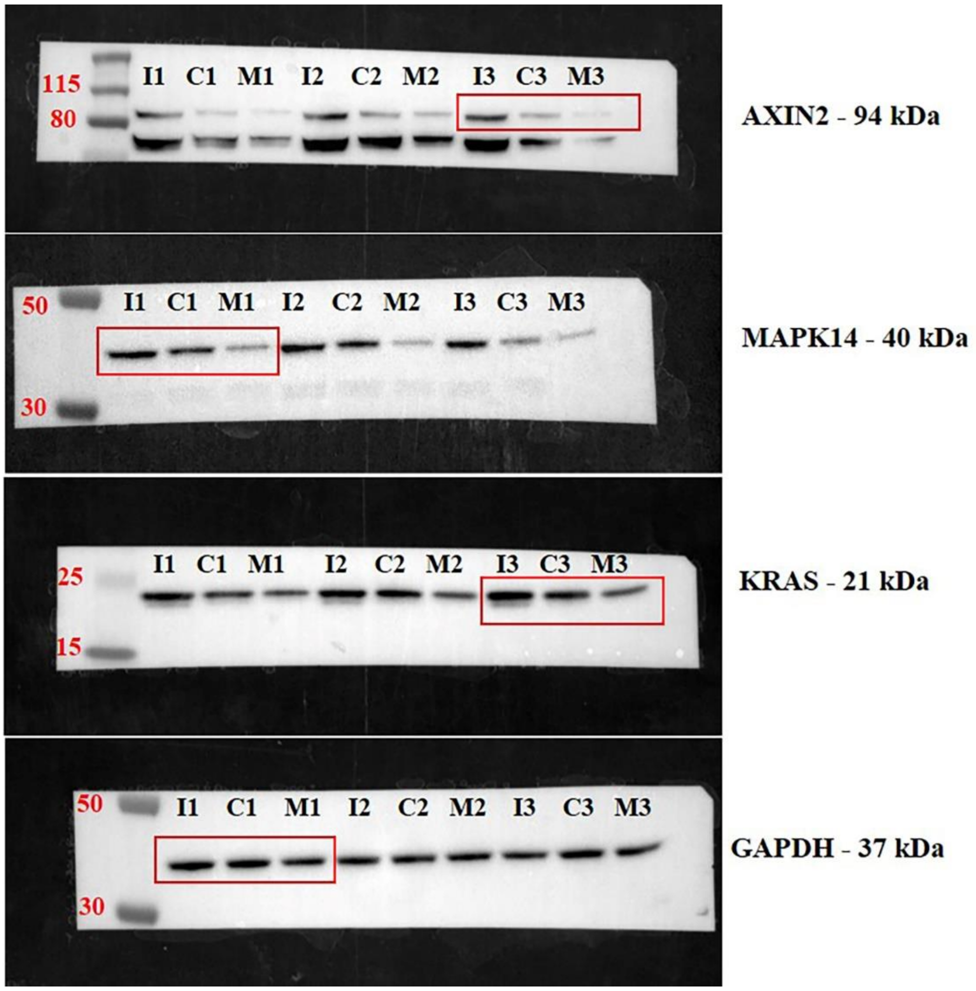

Fig. S2 a. Full length Western blots for Fig. 2 (AXIN2, MAPK14, KRAS)

The original Western blot images representing the protein expression in hsa-miR-143-3p transfected cells grown in 2D culture system. I-inhibitor treated group; C-transfection control group; M- mimic transfected group (n=3). 1, 2,3 represents the corresponding replicate. The region of the original blot used in Fig. 2 is denoted with red box.

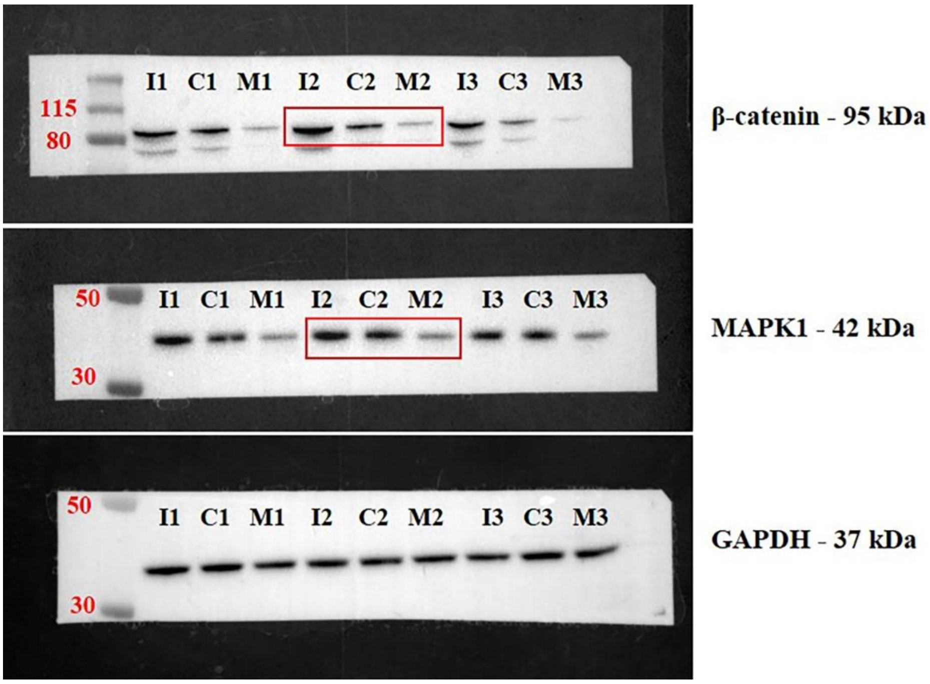

Fig. S2 b. Full length Western blots for Fig. 2 (β-catenin, MAPK1)

The original Western blot images representing the protein expression in hsa-miR-143-3p transfected cells grown in 2D culture system. I-inhibitor treated group; C-transfection control group; M- mimic transfected group (n=3). 1, 2,3 represents the corresponding replicate. The region of the original blot used in Fig. 2 is denoted with red box.

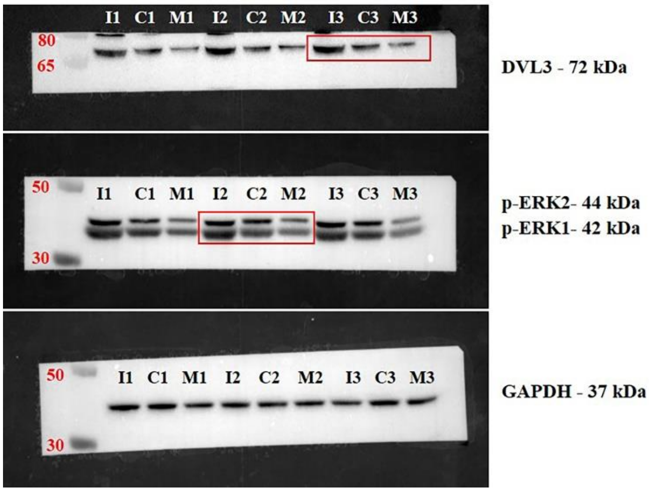

Fig. S2 c. Full length Western blots for Fig. 2 (DVL3, p-ERK1/2)

The original Western blot images representing the protein expression in hsa-miR-143-3p transfected cells grown in 2D culture system. I-inhibitor treated group; C-transfection control group; M- mimic transfected group (n=3). 1, 2,3 represents the corresponding replicate. The region of the original blot used in Fig. 2 is denoted with red box.

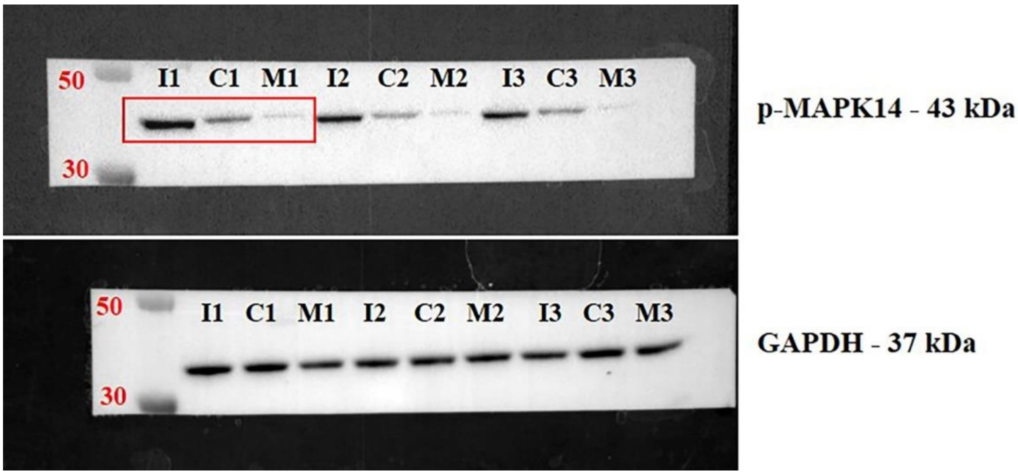

Fig. S2 d. Full length Western blots for Fig. 2 (p-MAPK14)

The original Western blot images representing the protein expression in hsa-miR-143-3p transfected cells grown in 2D culture system. I-inhibitor treated group; C-transfection control group; M- mimic transfected group (n=3). 1, 2,3 represents the corresponding replicate. The region of the original blot used in Fig. 2 is denoted with red box.

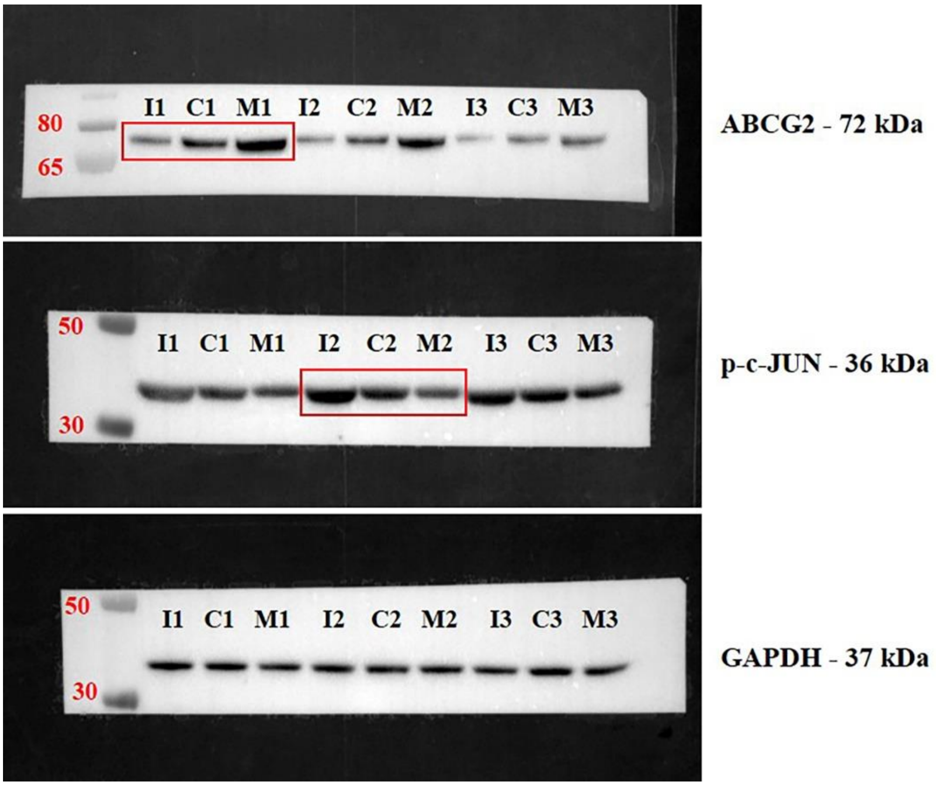

Fig. S2 e. Full length Western blots for Fig. 2 (ABCG2, p-c-JUN)

The original Western blot images representing the protein expression in hsa-miR-143-3p transfected cells grown in 2D culture system. I-inhibitor treated group; C-transfection control group; M- mimic transfected group (n=3). 1, 2,3 represents the corresponding replicate. The region of the original blot used in Fig. 2 is denoted with red box.

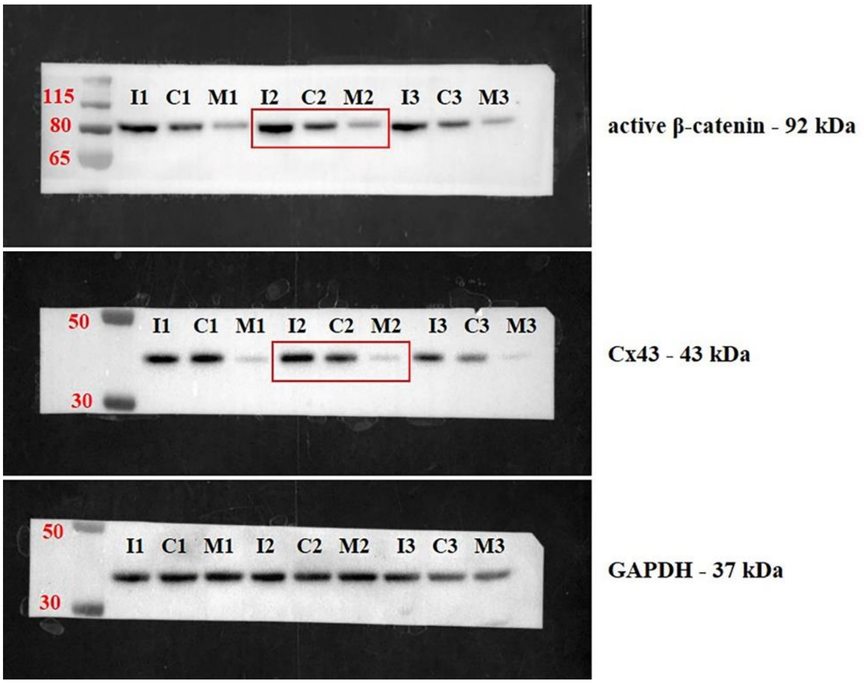

Fig. S2 f. Full length Western blots for Fig. 2 (active β-catenin, Cx43)

The original Western blot images representing the protein expression in hsa-miR-143-3p transfected cells grown in 2D culture system. I-inhibitor treated group; C-transfection control group; M- mimic transfected group (n=3). 1, 2,3 represents the corresponding replicate. The region of the original blot used in Fig. 2 is denoted with red box.

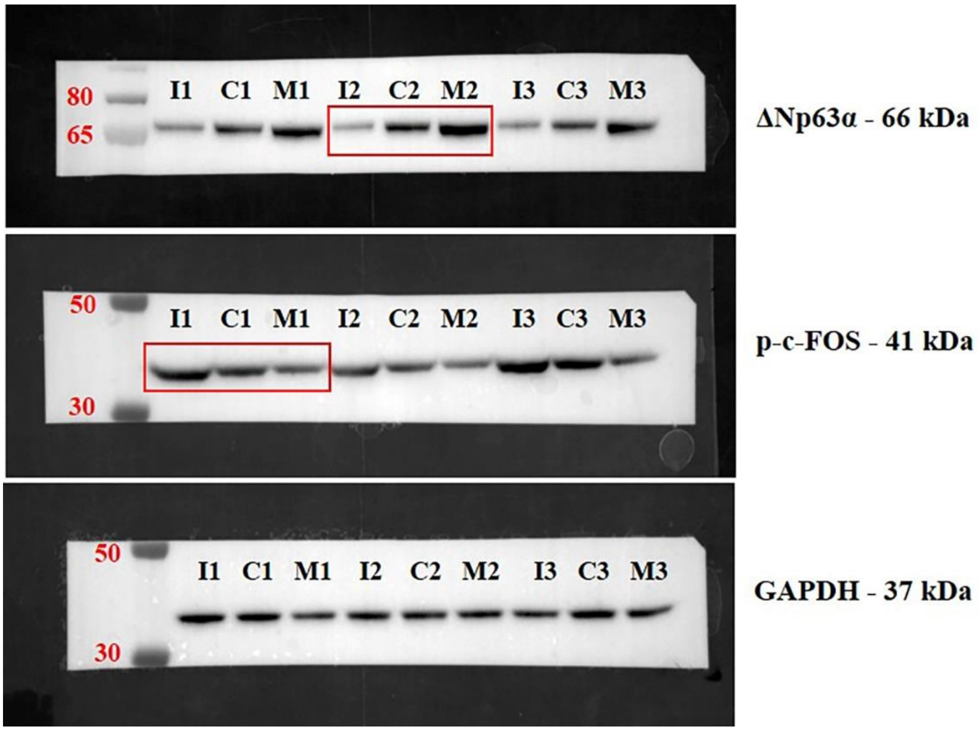

Fig. S2 g. Full length Western blots for Fig. 2 (ΔNp63α, p-c-FOS)

The original Western blot images representing the protein expression in hsa-miR-143-3p transfected cells grown in 2D culture system. I-inhibitor treated group; C-transfection control group; M- mimic transfected group (n=3). 1, 2,3 represents the corresponding replicate. The region of the original blot used in Fig. 2 is denoted with red box.

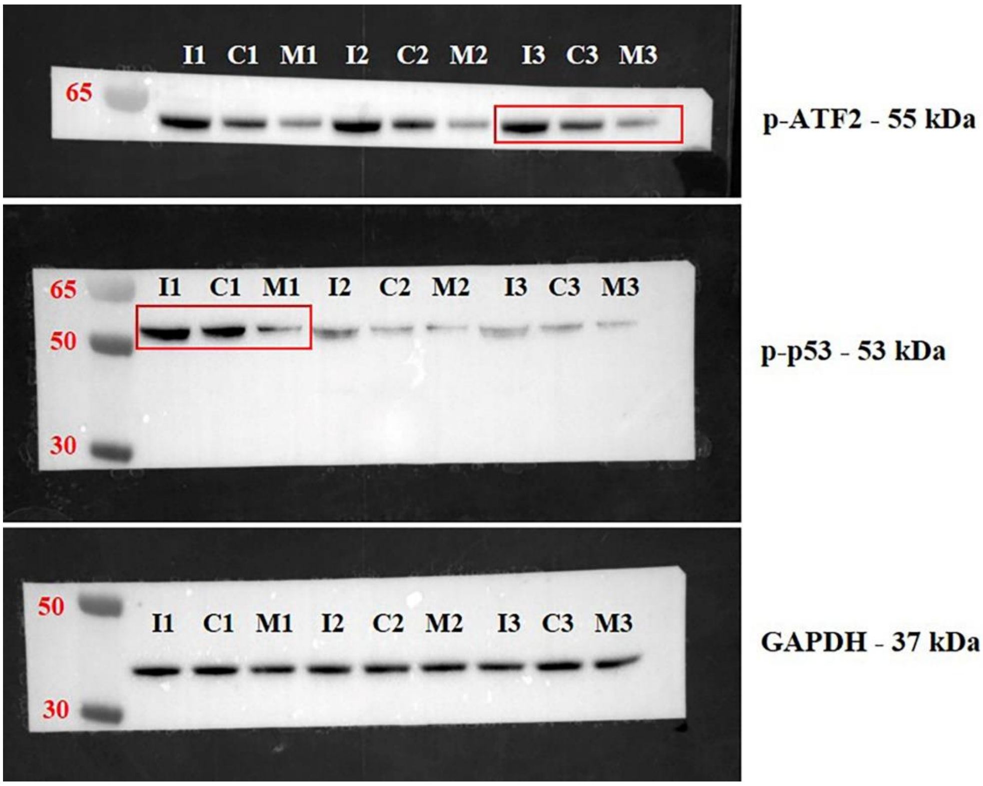

Fig. S2 h. Full length Western blots for Fig. 2 (p-ATF2, p-p53)

The original Western blot images representing the protein expression in hsa-miR-143-3p transfected cells grown in 2D culture system. I-inhibitor treated group; C-transfection control group; M- mimic transfected group (n=3). 1, 2,3 represents the corresponding replicate. The region of the original blot used in Fig. 2 is denoted with red box.

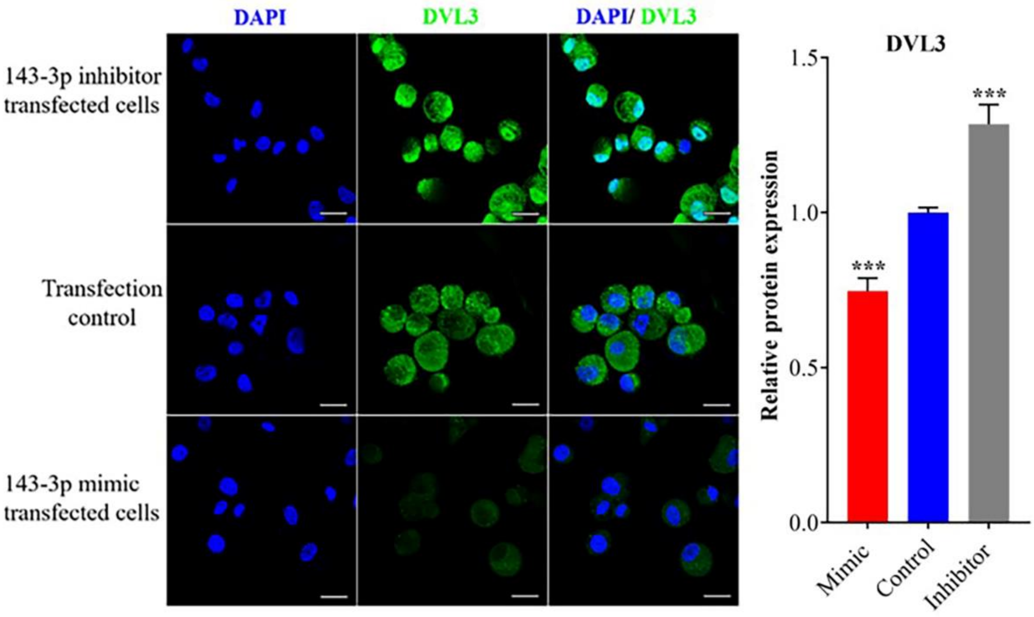

Fig. S3 a. Expression of DVL3 in hsa-miR-143-3p transfected cells

Representative immunostained confocal images of transfected primary limbal epithelial cells for DVL3 (Green) and nuclei (Blue). The expression of DVL3 was reduced in mimic transfected cells and increased in inhibitor transfected cells compared to control. Scale bar 50µm. The bar graph represents the relative protein expression based on fluorescence intensity in 3 groups i) control ii) mimic transfected group (Mimic) and iii) inhibitor transfected group (Inhibitor). Each sample (n=3) was analysed in triplicate and the data were expressed as mean ± SD. ***P < 0.0001

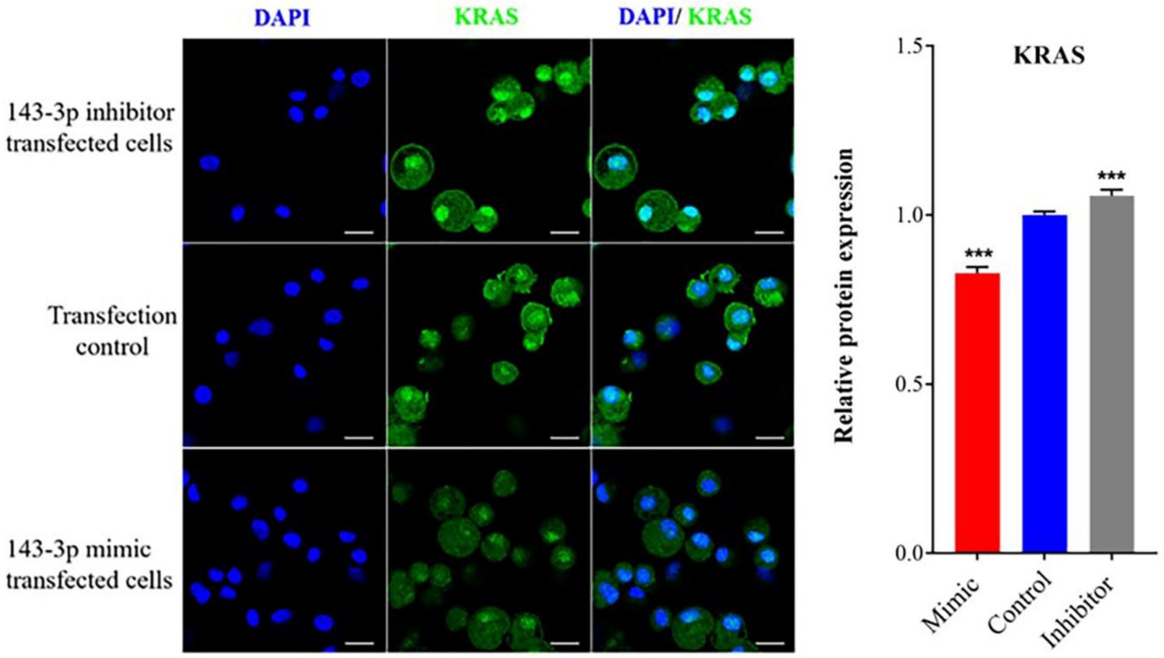

Fig. S3 b. Expression of KRAS in hsa-miR-143-3p transfected cells

Representative immunostained confocal images of transfected primary limbal epithelial cells for KRAS (Green) and nuclei (Blue). The expression of KRAS was reduced in mimic transfected cells and increased in inhibitor transfected cells compared to control. Scale bar 50µm. The bar graph represents the relative protein expression based on fluorescence intensity in 3 groups i) control ii) mimic transfected group (Mimic) and iii) inhibitor transfected group (Inhibitor). Each sample (n=3) was analysed in triplicate and the data were expressed as mean ± SD. ***P < 0.0001

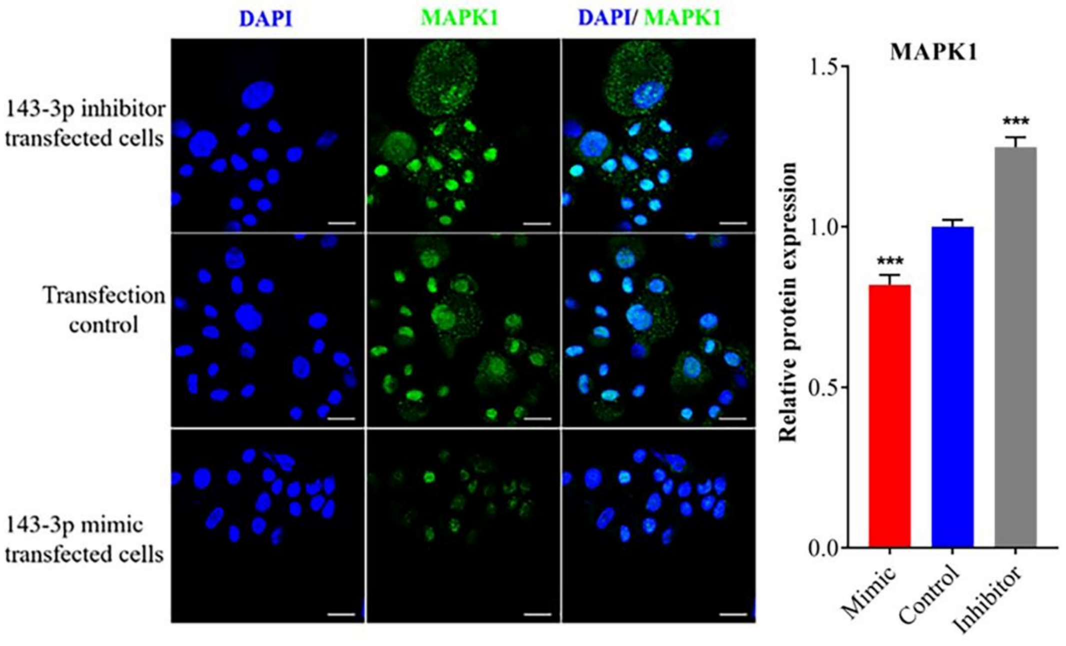

Fig. S3 c. Expression of MAPK1/ERK2 in hsa-miR-143-3p transfected cells

Representative immunostained confocal images of transfected primary limbal epithelial cells for MAPK1/ERK2 (Green) and nuclei (Blue). The expression of MAPK1/ERK2 was reduced in mimic transfected cells and increased in inhibitor transfected cells compared to control. Scale bar 50µm. The bar graph represents the relative protein expression based on fluorescence intensity in 3 groups i) control ii) mimic transfected group (Mimic) and iii) inhibitor transfected group (Inhibitor). Each sample (n=3) was analysed in triplicate and the data were expressed as mean ± SD. ***$P < 0.0001$

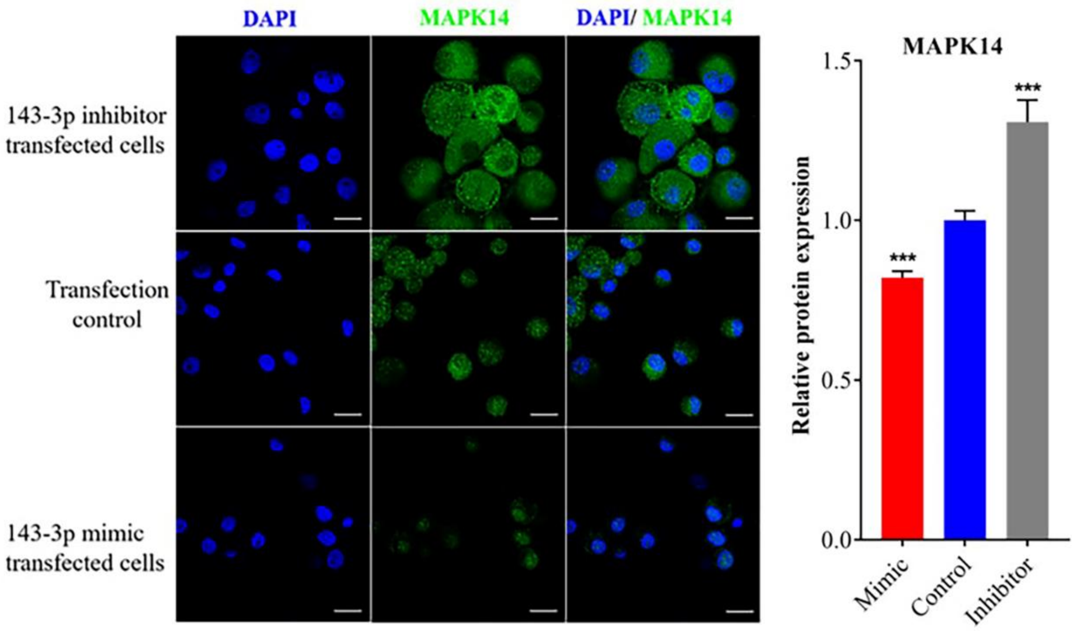

Fig. S3 d. Expression of MAPK14/p38 in hsa-miR-143-3p transfected cells

Representative immunostained confocal images of transfected primary limbal epithelial cells for MAPK14/p38 (Green) and nuclei (Blue). The expression of MAPK14/p38 was reduced in mimic transfected cells and increased in inhibitor transfected cells compared to control. Scale bar 50µm. The bar graph represents the relative protein expression based on fluorescence intensity in 3 groups i) control ii) mimic transfected group (Mimic) and iii) inhibitor transfected group (Inhibitor). Each sample (n=3) was analysed in triplicate and the data were expressed as mean ± SD. ***$P < 0.0001$

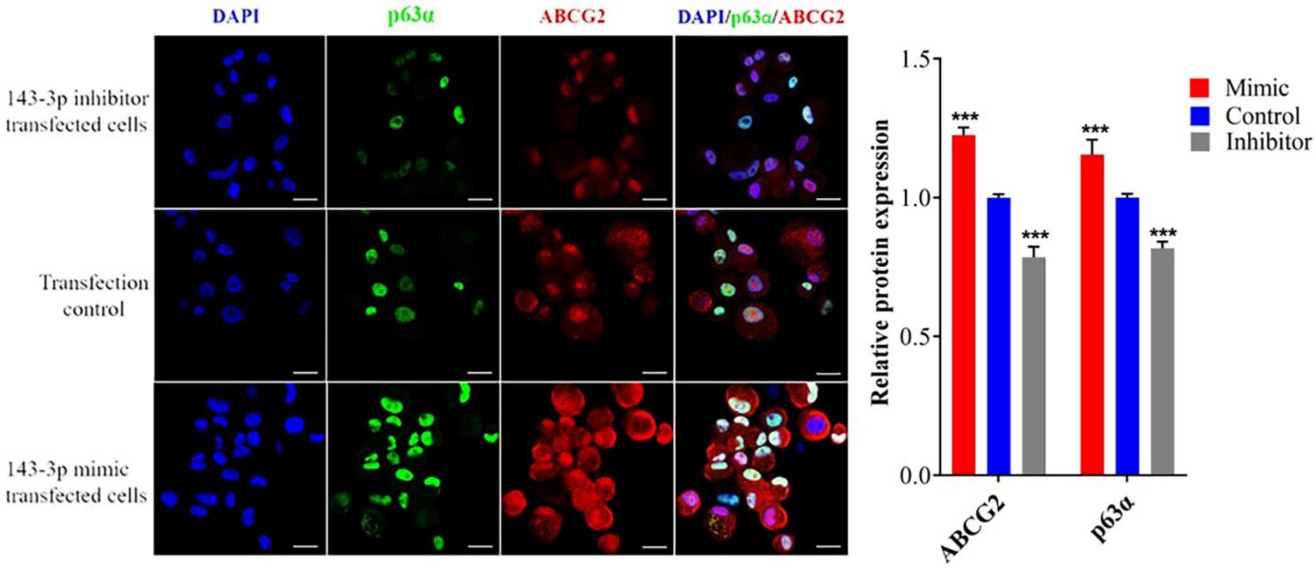

Fig. S3 e. Expression of ABCG2 and p63α in hsa-miR-143-3p transfected cells

Representative immunostained confocal images of transfected primary limbal epithelial cells for p63α (Green), ABCG2 (Red) and nuclei (Blue). The expression of p63α and ABCG2 was increased in mimic transfected cells and reduced in inhibitor transfected cells compared to control. Scale bar 50μm. The bar graph represents the relative protein expression based on fluorescence intensity in 3 groups i) control ii) mimic transfected group (Mimic) and iii) inhibitor transfected group (Inhibitor). Each sample (n=3) was analysed in triplicate and the data were expressed as mean ± SD. ****P* < 0.0001

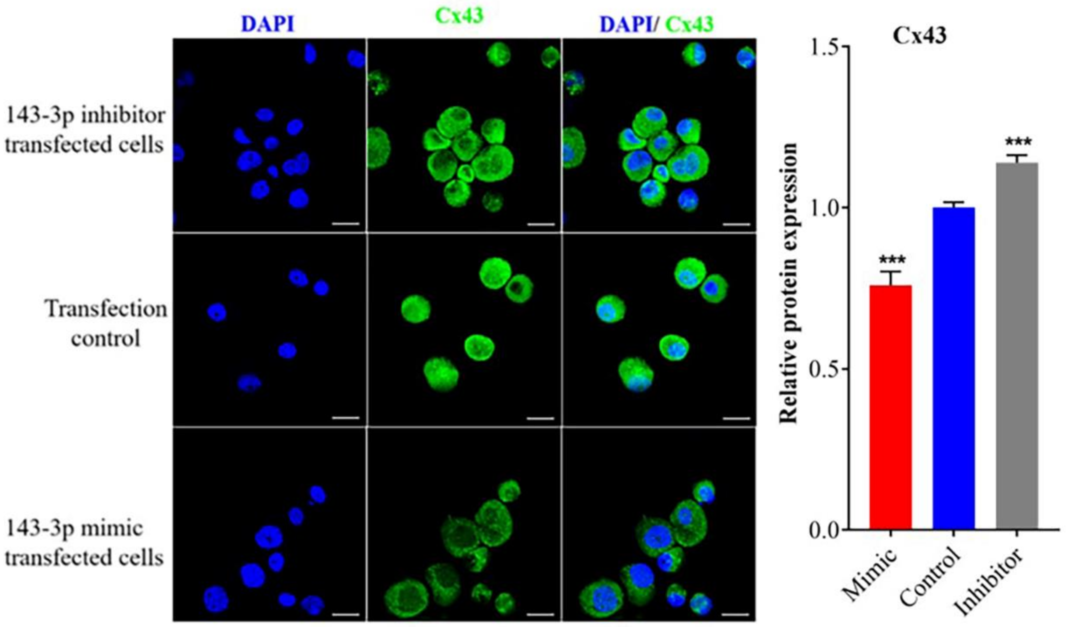

Fig. S3 f. Expression of Cx43 in hsa-miR-143-3p transfected cells

Representative immunostained confocal images of transfected primary limbal epithelial cells for Cx43 (Green) and nuclei (Blue). The expression of Cx43 was reduced in mimic transfected cells and increased in inhibitor transfected cells compared to control. Scale bar 50μm. The bar graph represents the relative protein expression based on fluorescence intensity in 3 groups i) control ii) mimic transfected group (Mimic) and iii) inhibitor transfected group (Inhibitor). Each sample (n=3) was analysed in triplicate and the data were expressed as mean ± SD. ****P* < 0.0001

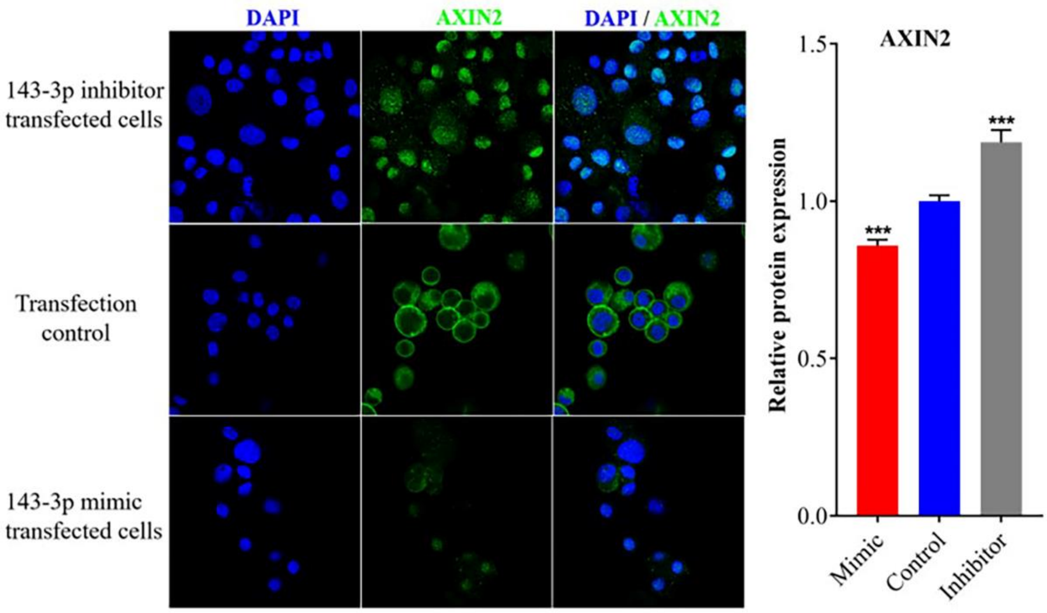

Fig. S3 g. Expression of AXIN2 in hsa-miR-143-3p transfected cells

Representative immunostained confocal images of transfected primary limbal epithelial cells for AXIN2 (Green) and nuclei (Blue). The expression of AXIN2 was reduced in mimic transfected cells and increased in inhibitor transfected cells compared to control. Scale bar 50µm. The bar graph represents the relative protein expression based on fluorescence intensity in 3 groups i) control ii) mimic transfected group (Mimic) and iii) inhibitor transfected group (Inhibitor). Each sample (n=3) was analysed in triplicate and the data were expressed as mean ± SD. ***$P <$ 0.0001

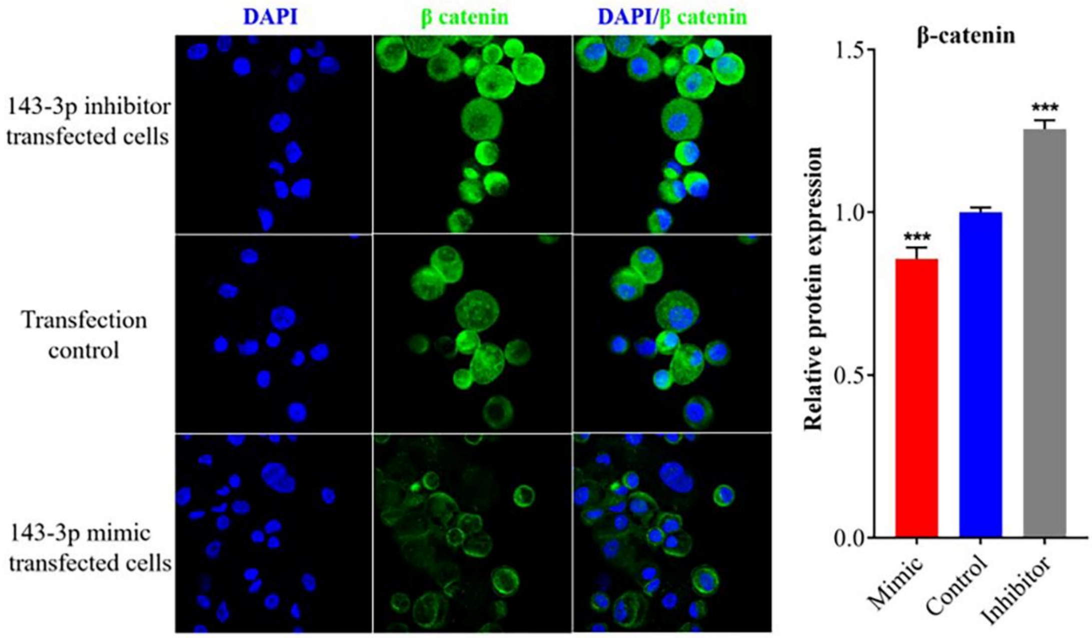

Fig. S3 h. Expression of β-catenin in hsa-miR-143-3p transfected cells

Representative immunostained confocal images of transfected primary limbal epithelial cells for β-catenin (Green) and nuclei (Blue). The expression of β-catenin was reduced in mimic transfected cells and increased in inhibitor transfected cells compared to control. The bar graph represents the relative protein expression based on fluorescence intensity in 3 groups i) control ii) mimic transfected group (Mimic) and iii) inhibitor transfected group (Inhibitor). Each sample (n=3) was analysed in triplicate and the data were expressed as mean ± SD. ***P < 0.0001

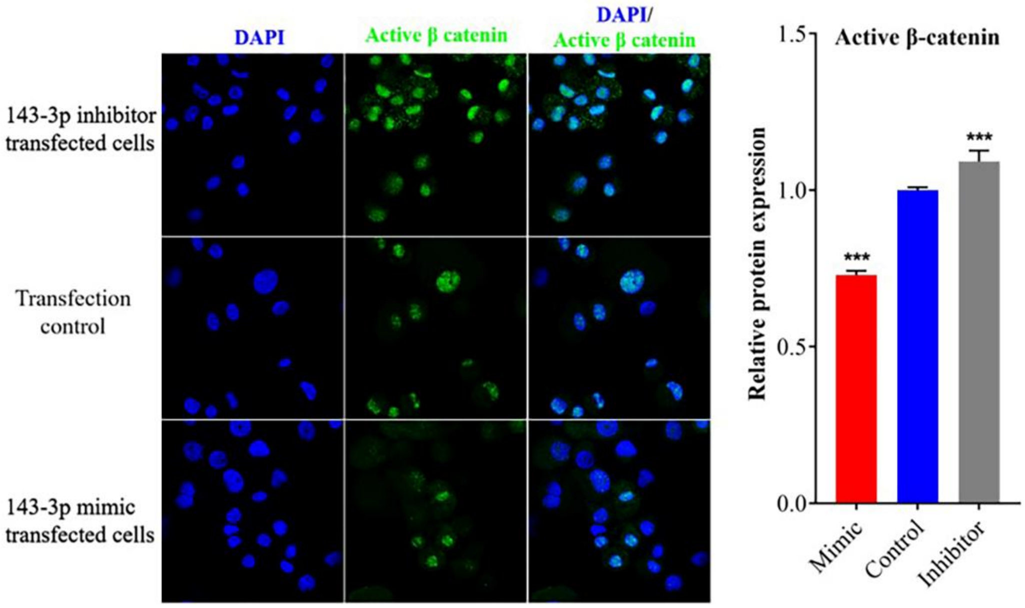

Fig. S3 i. Expression of active β-catenin in hsa-miR-143-3p transfected cells

Representative immunostained confocal images of transfected primary limbal epithelial cells for active β-catenin (Green) and nuclei (Blue). The expression of active β-catenin was reduced in mimic transfected cells and increased in inhibitor transfected cells compared to control. Scale bar 50µm. The bar graph represents the relative protein expression based on fluorescence intensity in 3 groups i) control ii) mimic transfected group (Mimic) and iii) inhibitor transfected group (Inhibitor). Each sample (n=3) was analysed in triplicate and the data were expressed as mean ± SD. ***P < 0.0001

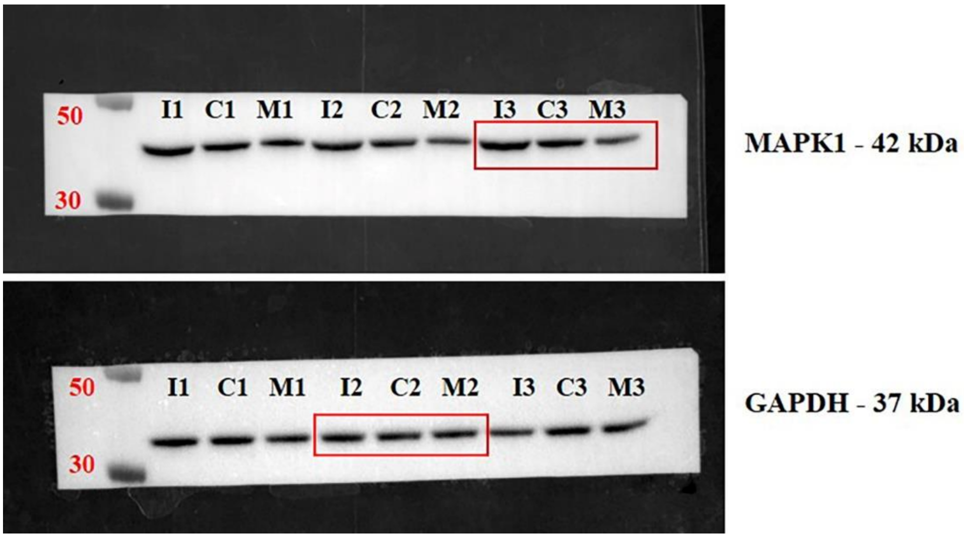

Fig. S4 a. Full length Western blots for Fig. 5 (MAPK1)

The original Western blot images representing the protein expression in hsa-miR-143-3p transfected cells grown in 3D RAFT culture system. I-inhibitor treated group; C-transfection control group; M-mimic transfected group (n=3). 1, 2,3 represents the corresponding replicate. The region of the original blot used in Fig. 5 is denoted with red box.

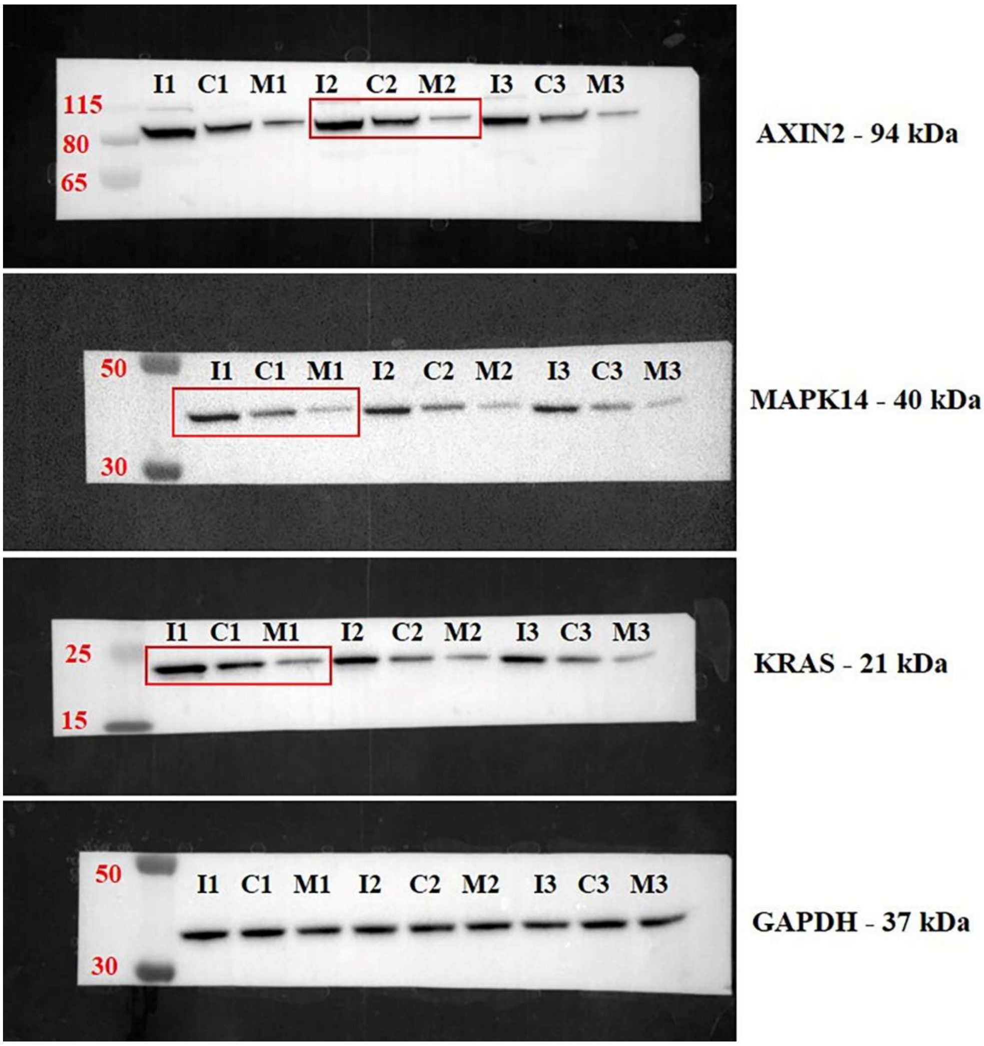

Fig. S4 b. Full length Western blots for Fig. 5 (AXIN2, MAPK14, KRAS)

The original Western blot images representing the protein expression in hsa-miR-143-3p transfected cells grown in 3D RAFT culture system. I-inhibitor treated group; C-transfection control group; M-mimic transfected group (n=3). 1, 2,3 represents the corresponding replicate. The region of the original blot used in Fig. 5 is denoted with red box.

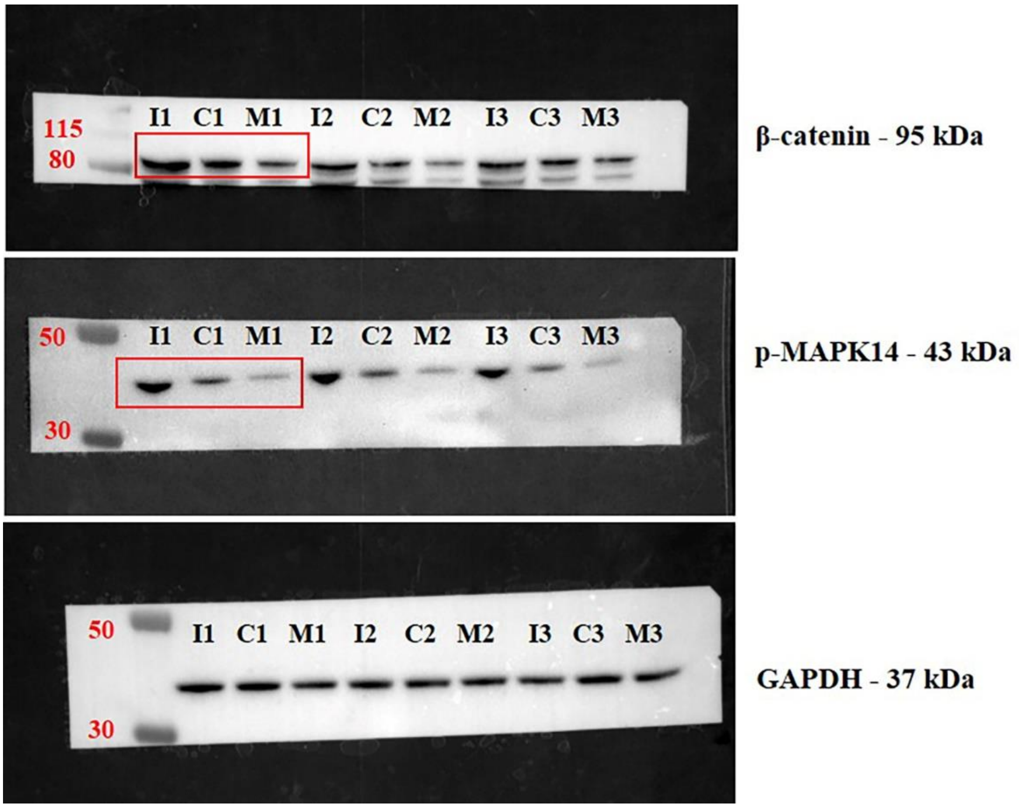

Fig. S4 c. Full length Western blots for Fig. 5 (β-catenin, p-MAPK14)

The original Western blot images representing the protein expression in hsa-miR-143-3p transfected cells grown in 3D RAFT culture system. I-inhibitor treated group; C-transfection control group; M-mimic transfected group (n=3). 1, 2,3 represents the corresponding replicate. The region of the original blot used in Fig. 5 is denoted with red box.

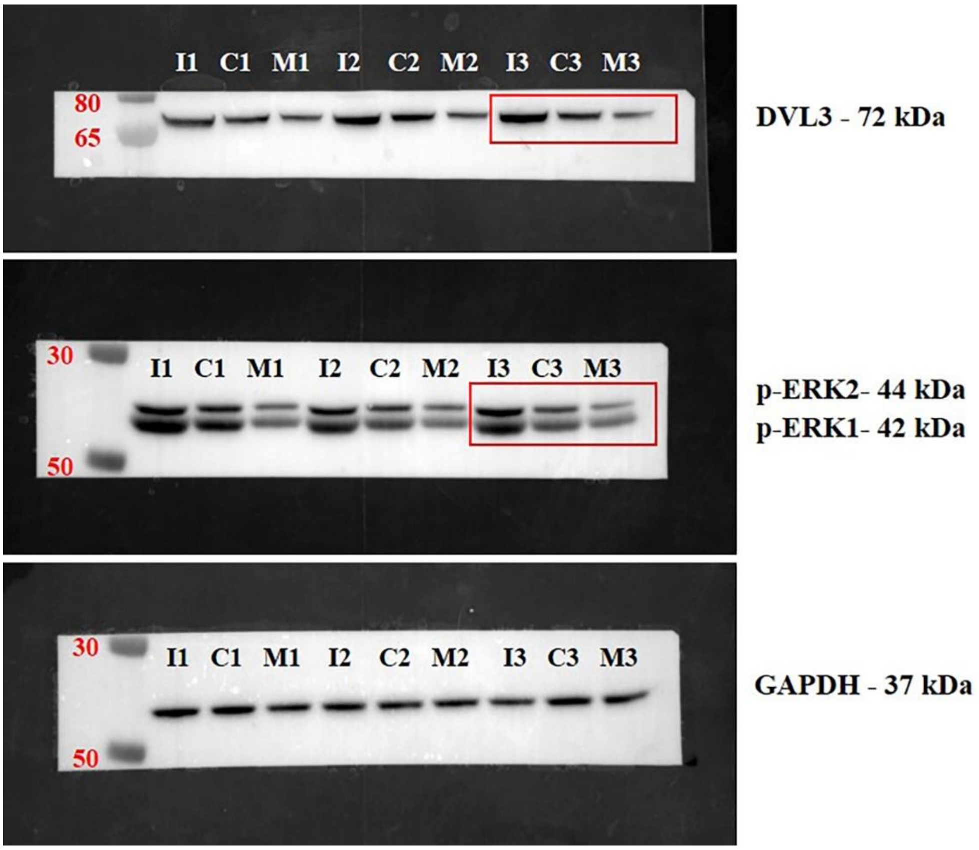

Fig. S4 d. Full length Western blots for Fig. 5 (DVL3, p-ERK1/2)

The original Western blot images representing the protein expression in hsa-miR-143-3p transfected cells grown in 3D RAFT culture system. I-inhibitor treated group; C-transfection control group; M-mimic transfected group (n=3). 1, 2,3 represents the corresponding replicate. The region of the original blot used in Fig. 5 is denoted with red box.

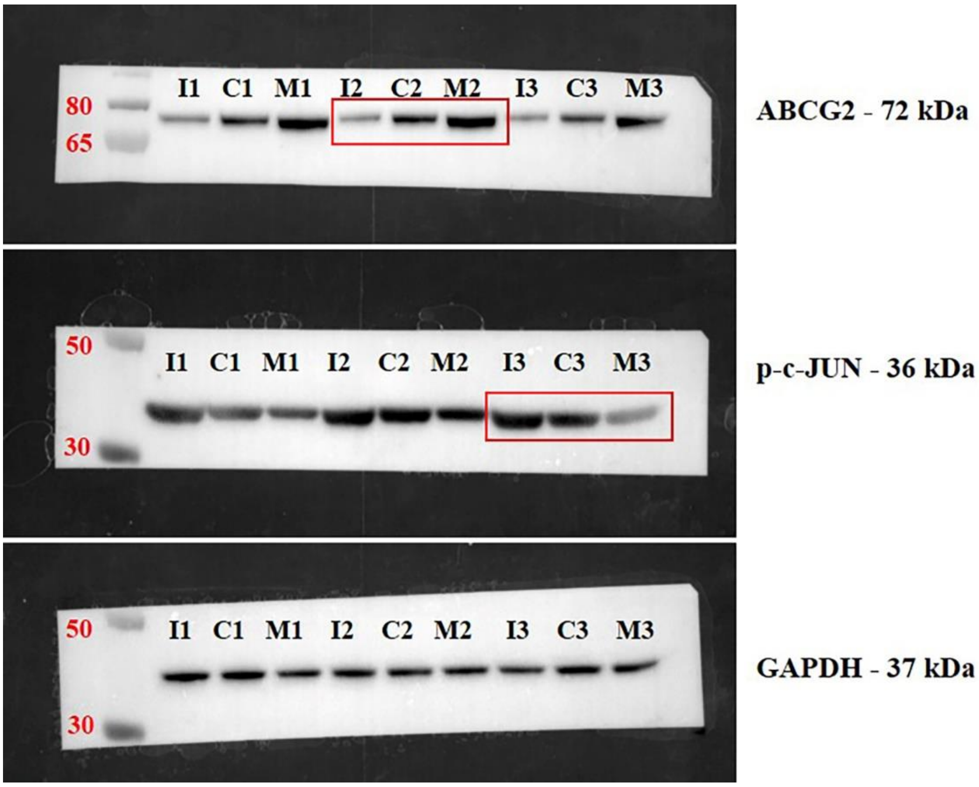

Fig. S4 e. Full length Western blots for Fig. 5 (ABCG2, p-c-JUN)

The original Western blot images representing the protein expression in hsa-miR-143-3p transfected cells grown in 3D RAFT culture system. I-inhibitor treated group; C-transfection control group; M-mimic transfected group (n=3). 1, 2,3 represents the corresponding replicate. The region of the original blot used in Fig. 5 is denoted with red box.

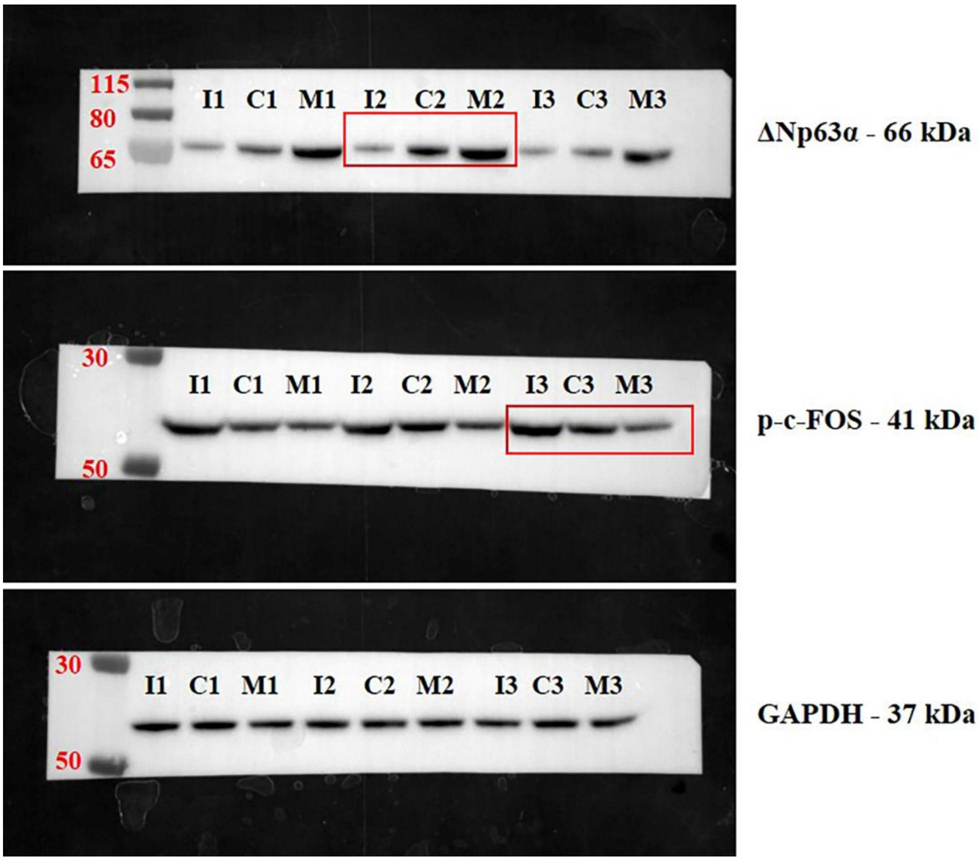

Fig. S4 f. Full length Western blots for Fig. 5 (ΔNp63α, p-c-FOS)

The original Western blot images representing the protein expression in hsa-miR-143-3p transfected cells grown in 3D RAFT culture system. I-inhibitor treated group; C-transfection control group; M-mimic transfected group (n=3). 1, 2,3 represents the corresponding replicate. The region of the original blot used in Fig. 5 is denoted with red box.

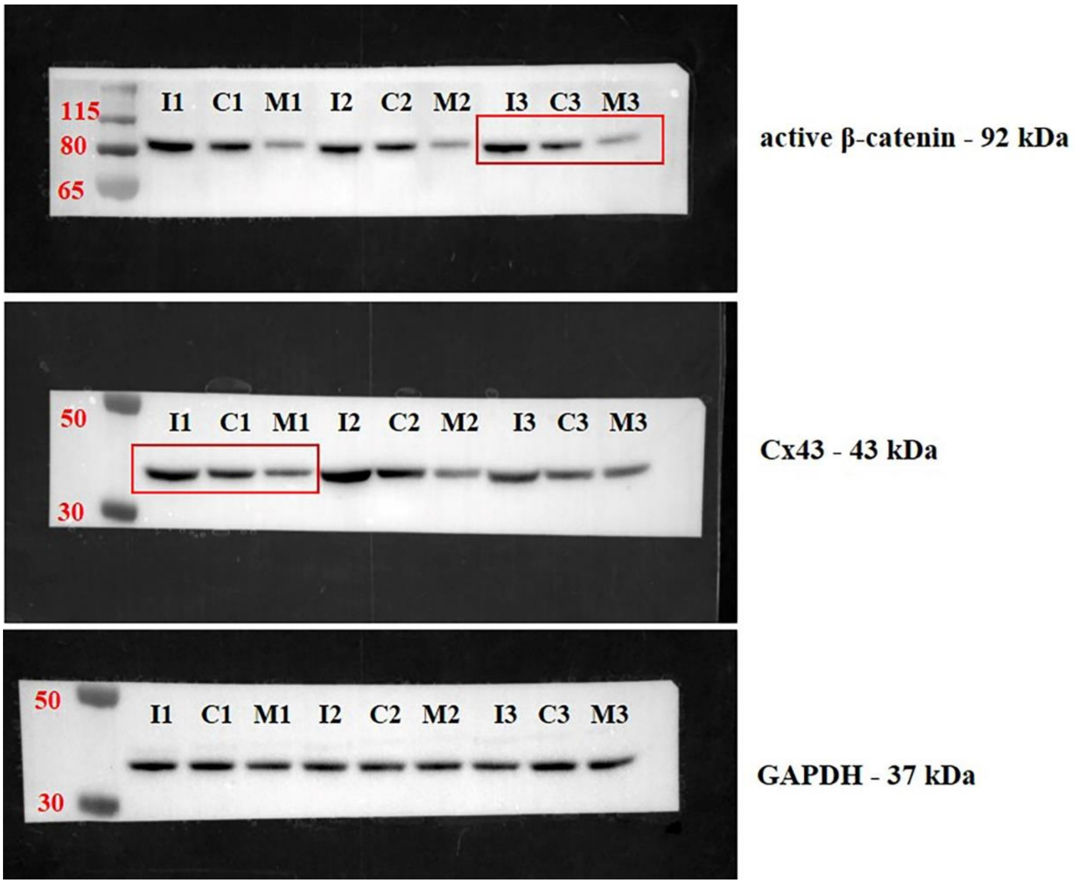

Fig. S4 g. Full length Western blots for Fig. 5 (active β-catenin, Cx43)

The original Western blot images representing the protein expression in hsa-miR-143-3p transfected cells grown in 3D RAFT culture system. I-inhibitor treated group; C-transfection control group; M-mimic transfected group (n=3). 1, 2,3 represents the corresponding replicate. The region of the original blot used in Fig. 5 is denoted with red box.

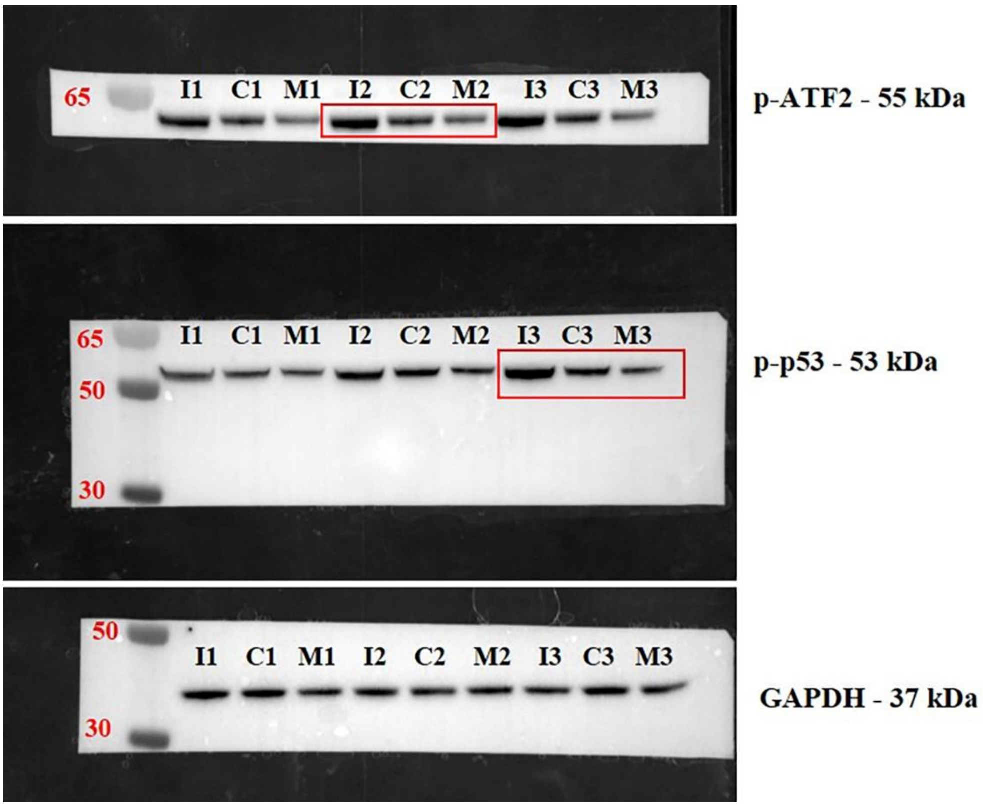

Fig. S4 h. Full length Western blots for Fig. 5 (p-ATF2, p-p53)

The original Western blot images representing the protein expression in hsa-miR-143-3p transfected cells grown in 3D RAFT culture system. I-inhibitor treated group; C-transfection control group; M-mimic transfected group (n=3). 1, 2,3 represents the corresponding replicate. The region of the original blot used in Fig. 5 is denoted with red box.

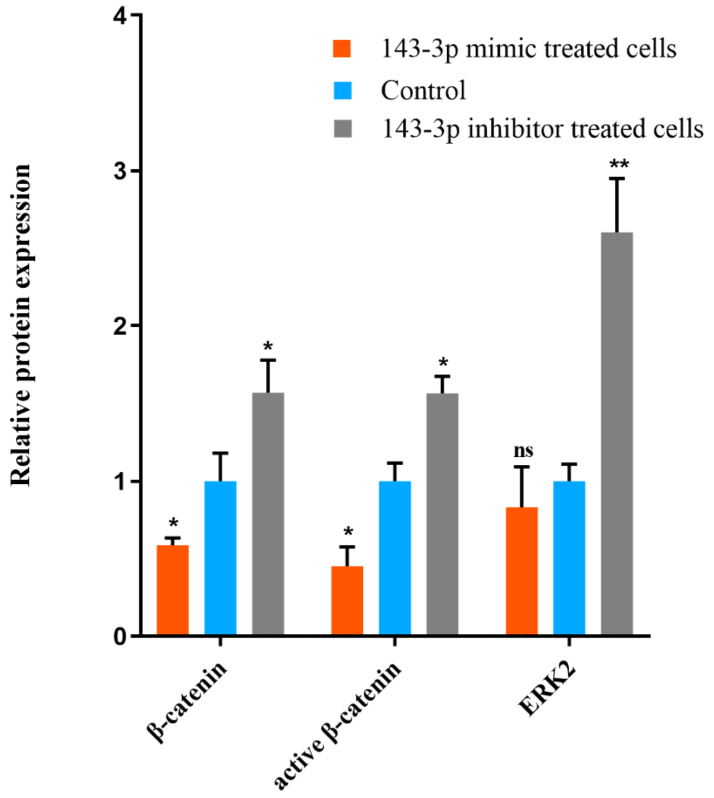

Fig. S5 Relative protein expression based on fluorescence intensity in transfected cells grown on 3D RAFT TEs

The bar graph represents the relative protein expression of β-catenin, active-β-catenin and ERK2 based on fluorescence intensity in 3 groups i) control ii) mimic transfected cells and iii) inhibitor transfected cells grown on 3D RAFT TEs. Each sample (n=3) was analysed in triplicate and the data were expressed as mean ± SD. **P* < 0.05; ***P* < 0.001; ns>0.05
